# An anchored experimental design and meta-analysis approach to address batch effects in large-scale metabolomics

**DOI:** 10.3389/fmolb.2022.930204

**Published:** 2022-11-09

**Authors:** Amanda O. Shaver, Brianna M. Garcia, Goncalo J. Gouveia, Alison M. Morse, Zihao Liu, Carter K. Asef, Ricardo M. Borges, Franklin E. Leach, Erik C. Andersen, I. Jonathan Amster, Facundo M. Fernández, Arthur S. Edison, Lauren M. McIntyre

**Affiliations:** ^1^ Department of Genetics, University of Georgia, Athens, GA, United States; ^2^ Complex Carbohydrate Research Center, University of Georgia, Athens, GA, United States; ^3^ Department of Chemistry, University of Georgia, Athens, GA, United States; ^4^ Department of Biochemistry, University of Georgia, Athens, GA, United States; ^5^ Department of Molecular Genetics and Microbiology, University of Florida, Gainesville, FL, United States; ^6^ School of Chemistry and Biochemistry, Georgia Institute of Technology, Atlanta, GA, United States; ^7^ Walter Mors Institute of Research on Natural Products, Federal University of Rio de Janeiro, Rio de Janeiro, Brazil; ^8^ Department of Environmental Health Science, University of Georgia, Athens, GA, United States; ^9^ Department of Molecular Biosciences, Northwestern University, Evanston, IL, United States; ^10^ University of Florida Genetics Institute, University of Florida, Gainesville, FL, United States

**Keywords:** untargeted metabolomics, meta-analysis, model organism, experimental design, nuclear magnetic resonance spectroscopy, mass spectrometry, batch effects

## Abstract

Untargeted metabolomics studies are unbiased but identifying the same feature across studies is complicated by environmental variation, batch effects, and instrument variability. Ideally, several studies that assay the same set of metabolic features would be used to select recurring features to pursue for identification. Here, we developed an anchored experimental design. This generalizable approach enabled us to integrate three genetic studies consisting of 14 test strains of *Caenorhabditis elegans* prior to the compound identification process*.* An anchor strain, PD1074, was included in every sample collection, resulting in a large set of biological replicates of a genetically identical strain that anchored each study. This enables us to estimate treatment effects within each batch and apply straightforward meta-analytic approaches to combine treatment effects across batches without the need for estimation of batch effects and complex normalization strategies. We collected 104 test samples for three genetic studies across six batches to produce five analytical datasets from two complementary technologies commonly used in untargeted metabolomics. Here, we use the model system *C. elegans* to demonstrate that an augmented design combined with experimental blocks and other metabolomic QC approaches can be used to anchor studies and enable comparisons of stable spectral features across time without the need for compound identification. This approach is generalizable to systems where the same genotype can be assayed in multiple environments and provides biologically relevant features for downstream compound identification efforts. All methods are included in the newest release of the publicly available SECIMTools based on the open-source Galaxy platform.

## Introduction

Untargeted metabolomics studies compare the variation in metabolites caused by genetic perturbations, treatments, and environmental differences (Liu and Locasale, 2017). Metabolomics is a powerful tool in biomarker discovery and holds great promise for precision medicine (Peng et al., 2015; Burgess, 2021; Schmidt et al., 2021). Targeted metabolomics is common in studies exploring human health questions that range from aging (Jones et al., 2012; Hastings et al., 2019) to complex diseases (Lewis et al., 2008; van der Sijde et al., 2014; Menni et al., 2017; Barupal et al., 2018; Rahman et al., 2021; Sindelar et al., 2021). An advantage of untargeted metabolomics for these questions is the ability to reach beyond sets of well-studied compounds to explore differences in an unbiased way (Cajka and Fiehn, 2016). Despite the attractiveness of an unbiased survey, untargeted metabolomics has challenges. In particular, the collection of highly variable biological material in a reproducible manner across batches makes the identification of differential compounds and comparisons of their abundances across datasets challenging. Chemical annotation of compounds, which is key to combining data across studies, requires considerable time and labor (Blazenovic et al., 2018). Given this bottleneck, it is essential to find novel ways to prioritize spectral features and overcome intractable challenges such as matrix effects, instrument drift, and batch variation (Sherman et al., 2018; Fan et al., 2019; Misra, 2020; Kim et al., 2021).

Batch effects across experiments are an enormous problem in untargeted metabolomics and a barrier to adopting these methods (De Livera et al., 2015). Normalizing to a quality control (QC) or biological reference material (BRM) included in each batch has been shown to be effective (Barupal et al., 2018; Fan et al., 2019; Kim et al., 2021). Although normalization strategies are improving (Fan et al., 2019; Kim et al., 2021); non-linear effects (Huaxu Yu and Huan, 2022), sample variation, the inability to separate environmental variance, and analytical artifacts (Misra, 2020) still pose ongoing challenges to the identification of common spectral features across studies. While different approaches to sample-based and data-based normalization have been described, such as total protein content, total ion count (TIC), and pooled QCs (Wulff JEM and Mitchell, 2018; Sindelar et al., 2021), reproducibility and heteroscedasticity (unequal variance) issues remain (Sumner et al., 2007; Fiehn et al., 2008; Dunn et al., 2012; Spicer et al., 2017; Broadhurst et al., 2018).

Our goal is to compare the same features across large numbers of independent samples (Smirnoff, 2018; Fang and Luo, 2019; Molon et al., 2020; Helf et al., 2022). As sample size increases, challenges associated with variation must be accounted for appropriately. In metabolomics studies, variation in pre-analytical sample collection (growth), analytical sample preparation (extraction), and data collection (instrument) (Gouveia et al., 2021) can be confounded. Identification of shared spectral features using a BRM is a successful strategy (Liu et al., 2020a; Gouveia et al., 2021) that has proven essential in large-scale studies (Beisken et al., 2015; Liu et al., 2020a; Wasito et al., 2021). Implementation of BRM controls for instrument variation can be used to estimate and normalize extraction variation (Sherman et al., 2018; Gouveia et al., 2021; Kim et al., 2021). In both liquid chromatography-mass spectrometry (LC-MS) and nuclear magnetic resonance (NMR) spectroscopy, ambiguity in whether features are generated by genetic or environmental factors coupled with batch effects and challenges in peak picking algorithms present obstacles to applying untargeted metabolomics to broader studies (Schrimpe-Rutledge et al., 2016; Misra, 2020). Although corrections for extraction and instrumentation variation exist, their utility in large studies for samples with complex matrices is limited (Beisken et al., 2015; Schrimpe-Rutledge et al., 2016; Chamberlain et al., 2019). Here, we use the model system *Caenorhabditis elegans* to demonstrate that an augmented design combined with experimental blocks (Federer WTaS and Schlottfeldt, 1954; Federer and Zelen, 1966; Federer et al., 2001) can be combined with other metabolomic QC approaches to anchor studies and enable comparisons of stable spectral features.

*C. elegans* is a model organism ideally suited to study conserved small molecules in metabolism (C. elegans Sequencing Consortium, 1998; Cook et al., 2017; Edison et al., 2016). The worm’s short life cycle, self-fertilization of homozygous hermaphroditic individuals, ease of cultivation, and ability to propagate large numbers of animals (Shaver et al., 2021) are ideal for large-scale studies (Hodgkin, 2001; Girard et al., 2007; Edison et al., 2016; Yilmaz and Walhout, 2016). These traits allow one to 1) develop, test, and validate approaches to identify stable spectral features, 2) demonstrate the feasibility of large-scale biochemical pathway analyses with genetic mutants, and 3) focus on spectral features likely to reveal essential components of metabolic pathways by comparing features that vary due to genetic perturbations.

**FIGURE 1:**
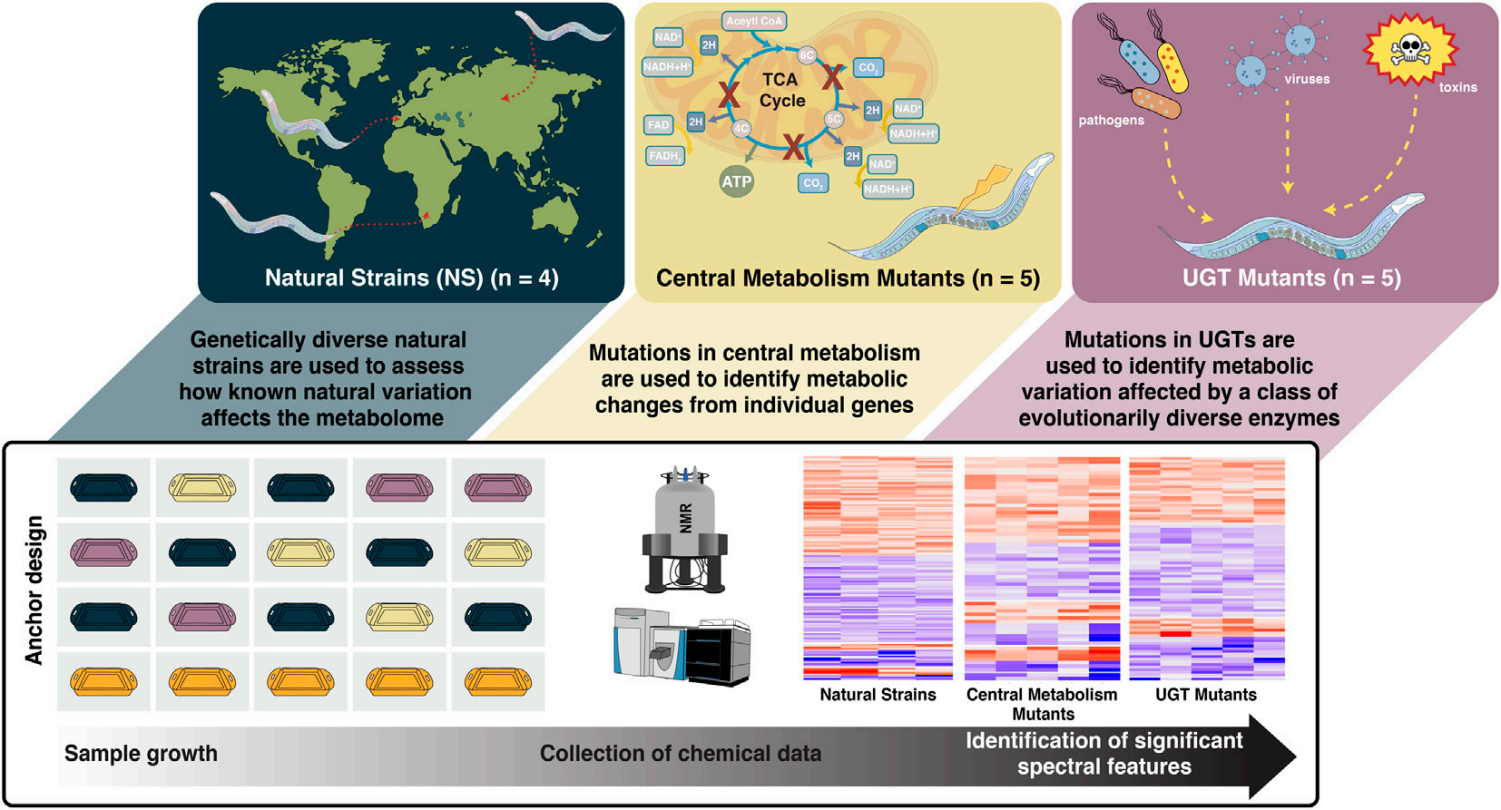
Fourteen *C. elegans* strains are evaluated in three genetic studies (natural strains, central metabolism mutants, and UDP- glucuronosyltransferase mutants). PD1074, the anchor control strain (orange), is grown alongside test strains (green, yellow, purple). Multiple biological replicates of PD1074 capture environmental variation in growth conditions. Non-polar and polar metabolic data were collected by nuclear magnetic resonance (NMR) spectroscopy and liquid chromatography-mass spectrometry (LC-MS). Data acquisition controls included biological reference material, pooled PD1074, pooled test strains, and extraction blanks. Biological replicates of PD1074 (n = 42 for LC-MS, n = 52 for NMR) were assayed individually and allocated across all data acquisition batches. Meta-analysis between PD1074 and individual test strains provided comparable inferences to mixed effects models, and the resulting estimated relative effects of each test strain to PD1074 provide straightforward comparisons of test strains between studies.

We designed three *C. elegans* studies to link natural and deliberate knock-out genetic perturbations (Figure 1). The first and second studies comprised central metabolism (CM) mutants and UDP-glucuronosyltransferase (UGT) mutants as examples of primary and secondary metabolism, respectively. CM mutants have been used in studies showing that diagnostic changes can be associated with human disease (Marquez et al., 2019; Martinez-Reyes and Chandel, 2020). UGTs are an evolutionarily diverse class of Phase 2 enzymes involved in detoxification (Yang et al., 2017; Meech et al., 2019). Although UGTs are vital to internal detoxification across species, the functions of UGTs have not been well described (Hasegawa et al., 2010; Stupp et al., 2013; Yang et al., 2017; Meech et al., 2019). The third study comprises genetically diverse natural strains (NS) from a broad geographic base, used to describe natural variation in the metabolome of *C. elegans* (Rockman and Kruglyak, 2006), including N2, a widely used laboratory-adapted strain (Zhang et al., 2021).

Collectively, CM and UGT mutants, and NS, allow us to 1) identify spectral features that vary due to genetic perturbations, 2) compare the same spectral features across all three studies without compound identification, and 3) plan future experiments that can be directly compared to these studies. The experimental design used here is straightforward to execute in model systems. An anchor strain (PD1074 here) is included alongside every test strain during growth and data collection, augmenting the design. Including the same strain enables the measurement and elimination of variation from non-genetic effects and the identification of stable features across a wide range of environmental conditions. Augmented designs are common in large-scale agricultural studies and are commonly used to compare large numbers of genotypes across heterogeneous environments (Federer WTaS and Schlottfeldt, 1954; Federer and Zelen, 1966). One of the main benefits of augmentation is the ability to estimate treatment effects within batches, thereby enabling the investigator to combine treatment effect estimates across batches using a relatively simple meta-analytic approach. This then avoids the complexities of estimating and adjusting for batch effects statistically. Given the limited resources and expense of compound identification, analysis of a set of stable spectral features for differences in intensity in several contexts provides one way of prioritizing compounds for identification.

## Methods

### *C. elegans* strain selection

This study used 15 *Caenorhabditis elegans* strains obtained from the *Caenorhabditis* Genetics Center (CGC) and *Caenorhabditis elegans* Natural Diversity Resource (CeNDR) (Cook et al., 2017). Fourteen *C. elegans* strains were used as ‘test strains’, and one strain, PD1074, was used as the anchor/reference strain (Supplementary Table S1). Strains were selected to cover the diversity of interests in the metabolomics community, including samples with mutations in primary and secondary metabolism and natural strains. PD1074 was selected as the anchor strain as it is a traceable variant of the laboratory-adapted N2 Bristol strain.

#### *C. elegans* sample growth and preparation

**FIGURE 2:**
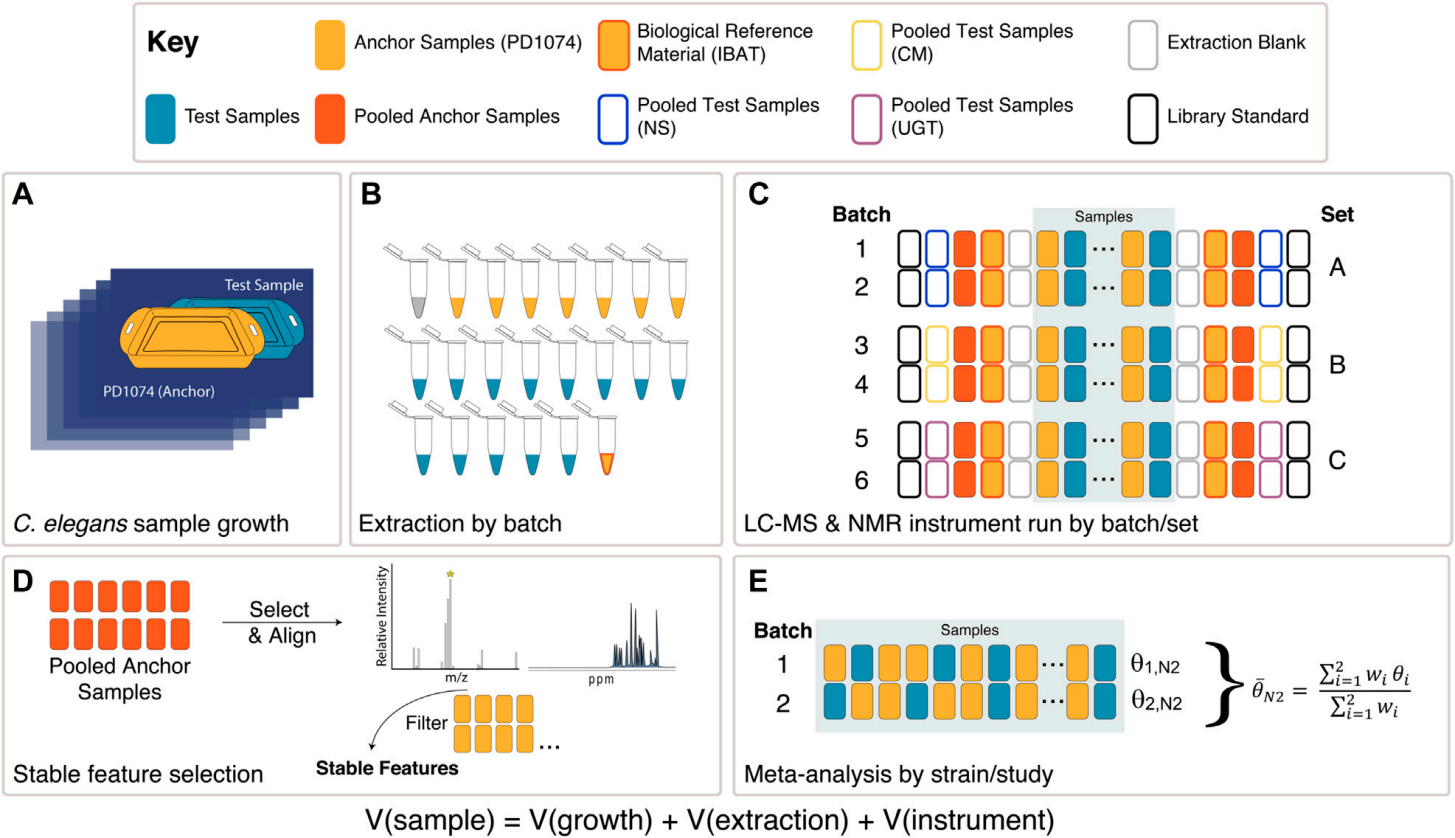
Experimental design overview. **(A)** Each *C. elegans* LSCP was grown and harvested with at least one PD1074 sample (sample growth variation captured) (Supplementary Figure S1). **(B)** PD1074 samples and test strains (NS, CM mutants, or UGT mutants), IBAT references, and extraction blanks were included in each batch for LC-MS or NMR (batch preparation variation captured). **(C)** A total of six batches in three sets were collected. Instrument controls, library standards, and pooled PD1074 samples were included at the start and end of each run (instrumentation variation captured) were in each run. Independent samples from each test strain were collected in two independent sequential batches. **(D)** In LC-MS, PD1074 spectral features were identified from PD1074 pools and retained if they were present above the level of the extraction blank in 100% of the individual PD1074 spectra. In NMR, semi-automated peak-picking and binning were performed to extract peak heights and identify stable peaks present in PD1074 samples. **(E)** Meta-analysis models to identify differences between test strains and PD1074.

Populations of genetically identical nematodes were independently generated for every biological replicate using a large-scale culture plate (LSCP) method (Shaver et al., 2021). *Escherichia coli* from an Iterative Batch Averaging Method (IBAT) was used as a food source for *C. elegans* strains grown on LSCPs (Gouveia et al., 2021). For each independent LSCP, nematodes were collected, population size estimated, and the sample was divided into at least 12 identical aliquots of 200,000 nematodes in ddH_2_O and flash-frozen in liquid nitrogen to quench metabolism and stored at −80°C (Shaver et al., 2021). We continuously seeded and harvested PD1074 as test samples were seeded or harvested during the LSCP growth process (Figure 2A, Supplementary Figure S1) (Shaver et al., 2021). PD1074 samples were included in each extraction batch (Figure 2B), and data were collected on multiple biological replicates concurrent with test samples (Figure 2C). These PD1074 samples anchor the three studies in an augmented design (Federer WTaS and Schlottfeldt, 1954; Federer et al., 2001).

### Iterative batch averaging method (IBAT) of PD1074

An IBAT *C. elegans* control for the PD1074 strain was included in the experimental design. Sequential aliquots of independently grown PD1074 were pooled together to generate a biological reference material (BRM) (Gouveia et al., 2021). While we used PD1074, any strain of *C. elegans* could serve this purpose. IBAT controls are used to estimate extraction variance and assist in alignment across batches (Figure 2B). All IBAT controls were generated independently from this experiment.

### Batch design

Centrifuge capacity dictated that extraction batches were capped at 24 samples. Six extraction batches were needed (104 test strains, Supplementary Table S1). Extraction batches included IBAT controls, extraction blanks, half of the biological replicates for each test sample, and four to seven PD1074 biological replicates. Extraction blanks control for homogenization and extraction steps to account for and remove non-biologically related LC-MS or NMR features that represent background effects. All NS were collected in batches 1 and 2. The CM mutants were collected in batches 3 and 4, with AUM2073 and VC2524 collected in batches 5 and 6. The UGT mutants were collected in batches 5 and 6, with RB2011 collected in batches 1 and 2. Batches were collected back-to-back in NMR but were collected over several months in LC-MS, although the column and instrument were unchanged. We note there was a needle failure between batches 5 and 6 in the HILIC LC-MS (+) run, and an instrument failure occurred during the collection of the HILIC negative data precluding us from including data on that fraction. For both NMR and LC-MS, library standards and batch pools for the PD1074 samples and the test strains were added to each run before data acquisition at the beginning and end of each batch (Figure 2C).

### NMR sample homogenization and extraction

Frozen lyophilized *C. elegans* aliquots were retrieved from -80°C. 200 μl of 1 mm zirconia beads (BioSpec Products) were added to each sample and homogenized at 420 rcf for 90 s in a FastPrep-96 homogenizer and subsequently placed on dry ice for 90 s to avoid overheating; this step was repeated twice.

Using the homogenized samples, 1 ml of 100% isopropanol (IPA) chilled to -20°C was added to the lyophilized/homogenized sample powder and Zirconia beads in two increments of 500 μl. After each addition of 500 μl, samples were vortexed for 30 s–1 min, and left at room temperature (RT) for 15–20 min. After RT incubation, samples were stored overnight (∼12 h) at -20°C. Samples were centrifuged for 30 min at 4°C (20,800 rcf). The supernatant was transferred to a new tube to analyze non-polar molecules. 1 ml of pre-chilled 80:20 methanol:water (CH_3_OH:H_2_O) (4°C) was added to the remaining nematode pellet to analyze polar molecules. The polar fraction was shaken at 4 °C for 30 min. Samples were centrifuged at 20,800 rcf for 30 min at 4°C. The supernatant was transferred to a new tube to analyze polar molecules. Both polar and non-polar samples were placed in a Labconco Centrivap at RT and monitored until dry. Polar samples were reconstituted in D_2_O (99%, Cambridge Isotope Laboratories, Inc.) in a 100 mM sodium phosphate buffered solution with 0.11 mM sodium 2,2-dimethyl-2-silapentane-5-sulfonate (DSS-D6; 98%; Cambridge Isotope Laboratories, Inc.). Non-polar samples were reconstituted in CDCl_3_ (99.96%; Cambridge Isotope Laboratories, Inc.). Samples were vortexed until fully soluble, and 45 μl of each sample were transferred into 1.7 mm NMR tubes (Bruker SampleJet) for acquisition.

### NMR acquisition

To collect the polar fraction, one-dimensional (1D) ^1^H NMR spectra were acquired with a noesypr1d pulse sequence on a NEO 800 MHz Bruker NMR spectrometer equipped with a 1.7 mm TCI cryoprobe and a Bruker SampleJet autosampler cooled to 6°C. During acquisition, 32,768 complex data points were collected using 128 scans with two dummy scans. The spectral width was set to 15 ppm.

To collect the non-polar fraction, 1D ^1^H NMR spectra were acquired with a zg pulse sequence (zg30). During acquisition, 65,536 complex data points were collected using 64 scans with four dummy scans. The spectral width was set to 20.2 ppm.

Immediately after each 1D acquisition, a two-dimensional (2D) J-resolved spectrum was collected using the Bruker pulse program jresgpprqf. For both the polar and non-polar fractions, 8,192 and 40 points were collected using eight scans, four dummy scans, and spectral widths of 16 and 0.09 ppm, respectively.

For NMR metabolite annotation three 2D experiments 1H–1H TOCSY (dipsi2gppphzspr), 1H-13C HSQC (hsqcetgpsisp2.2) and 1H-13C HSQC-TOCSY (hsqcdietgpsisp.2) were collected on separate pooled PD1074 polar samples. The HSQC experiment was collected using 6,250 and 720 points in the indirect and direct dimensions, 32 scans and 16 dummy scans and a spectral width of 13 ppm for the proton and 165 ppm for the carbon dimensions. The HSQC-TOCSY experiment parameters were identical to HSQC except for 32 dummy scans and a 90 ms mixing time. The TOCSY experiment was collected with 7,272 points and 800 points in the indirect and direct dimensions, 32 scans and 16 dummy scans, a spectral width of 11.367 ppm in both dimensions and a DIPSI2 mixing time of 90 ms.

#### Reproducibility

LC-MS and NMR study design, sample collection, sample preparation, instrument parameters, and chromatographic data can be found on Metabolomics Workbench, https://www.metabolomicsworkbench.org Study ID (LC-MS: ST002092; NMR polar: ST002095; NMR non-polar: ST002096). Step-by-step guides for data processing with all individual scripts are available at: https://github.com/artedison/metaanalysis. Pre-processing steps, input parameters, and set values used for LC-MS data are also available in Supplementary Table S5. All data analysis scripts with detailed step-by-step documentation for each of the five technologies are provided https://github.com/McIntyre-Lab/papers/tree/master/shaver_metaanalysis_2022. For convenience, all data used in analyses, and all analysis results have also been compiled in Supplementary Data Sheet S2.

### NMR data processing and stable feature selection

Data were processed using NMRPipe (Delaglio et al., 1995) (Version: mac11_64, https://www.ibbr.umd.edu/nmrpipe). A Fourier transform with an exponential line broadening of 1.5 Hz, and manual phase correction were carried out (Delaglio et al., 1995). Using tools from MATLAB (The MathWorks, R2019a (MATLAB, 2019)), the spectra were referenced to 7.24 ppm using the CDCl_3_ resonance, and the polar extracts are referenced to 0.00 ppm using DSS. Solvent regions were removed followed by baseline correction using a statistical smoothing function (Xi and Rocke, 2008). Alignment was performed using CCOW (Tomasi and Andersson, 2004) and PAFFT (Wong et al., 2005) algorithms, and a binning algorithm was applied. PD1074 samples, PD1074 pools, and IBAT controls were visually compared, and peaks present in all samples were manually identified (ppm). A total of 589 and 575 stable spectral features were identified in the NMR polar and non-polar data, respectively.

The blank feature filtering (BFF) threshold (BFF_threshold_) was calculated using the extraction blanks (B) as shown in Eq. 1.

$${BFF}_{threshold}=B_{average}+3\times B_{standard\;deviation}$$ (1)

Individual features were retained if the average value of the feature for the PD1074 samples was more than 100 times greater than the threshold (Patterson et al., 2017). After BFF, a total of 585 in NMR polar and 487 in NMR non-polar stable spectral features were retained. Two-dimensional NMR experiments were also processed using NMRPipe (Delaglio et al., 1995). Spectra were Fourier transformed using a 90° shifted sine window function and automatic zero filling, manually phased, and referenced to DSS or CDCl_3_ (Supplementary Figure S2).

### LC-MS sample homogenization and extraction

Frozen aliquots of 200,000 *C. elegans* nematodes were retrieved from -80°C and lyophilized in a VirTis^®^ BenchTop™ “K” Series Freeze Dryer (*SP Industries, Inc.)*. Using glass and zirconium oxide beads, the aliquots were homogenized for 3 minutes in a Qiagen Tissuelyser 2. Homogenized nematodes were extracted with 1.5 ml of IPA at -20°C overnight (approximately 12 h), then pelleted and the supernatant transferred to separate 2 ml centrifuge tubes. Supernatants were dried to completion in a Labconco Centrivap and stored at -80°C for non-polar LC-MS analysis. The pellet was extracted a second time using 80:20 CH_3_OH:H_2_O (v:v) for 20 min at RT while shaking at 1,500 rpm. Samples were pelleted to separate proteins, and the supernatant was transferred to separate 2 ml centrifuge tubes, dried down to completion, and stored at -80°C for polar LC-MS analysis.

### LC-MS data acquisition

Non-polar extracts were reconstituted in 75 µl of IPA containing isotopically labeled lipid standards (a detailed list of standards is included in the SI) and analyzed by LC-MS using a ThermoFisher Scientific Accucore C30 150 × 2.1mm, 2.6 µm column paired with a Thermo Fisher Orbitrap ID-X in positive and negative polarity. Polar (80:20 CH_3_OH:H_2_O) extracts were reconstituted in 75 µl of 80:20 CH_3_OH:H_2_O containing isotopically labeled arginine, hypoxanthine, hippuric acid, and methionine (Cambridge Isotope Laboratories, Inc.) and analyzed by LC-MS using a Waters BEH Amide 150 × 2.1 mm, 1.7 µm column paired with a Thermo Fisher Orbitrap ID-X in positive and negative polarity. LC-MS/MS data for each mode of analysis was collected using three rounds of iterative DDA (Thermo Scientific AcquireX) performed on pooled test samples.

Data for each sample were collected in full MS1 with a resolution of 240,000 FWHM (full-width half-maximum) and MS/MS spectra of pooled samples were collected at a resolution of 30,000 FWHM using a 0.8 Da isolation window and stepped HCD collision energies of 15, 30, and 45.

### LC-MS data processing and stable feature selection

Thermo .raw files were converted to centroid mode and .mzML format using Proteowizard’s MSconvertGUI tool (Chambers et al., 2012). We used the memory-efficient large-scale pipeline SLAW (Delabriere, et al., 2021; https://github.com/zamboni-lab/SLAW) for parameter optimization and data processing. SLAW offers the following peak picking algorithms: XCMS centWave (Smith et al., 2006; Tautenhahn et al., 2008), OpenMS FeatureFinderMetabo (Kenar et al., 2014; Röst et al., 2016), and MZmine ADAP (Pluskal et al., 2010; Myers et al., 2017) For this study, ADAP was selected (Myers et al., 2017). ADAP parameter optimization was carried out using the 12 PD1074 pools included in each batch, and features were retained if present above the noise threshold in all PD1074 pools. We then selected features using the same BFF algorithm described for the NMR data processing in Eq. 1 (Patterson et al., 2017) and removed solvent front features based on the retention time of the void volume. Features were retained if present in 100% of the PD1074 spectra (pools and individual samples).

LC-MS spectral features often vary across biological replicates. Additional complexities include retention time drift, batch effects, and algorithmic limitations in estimating peak abundances in complex spectra (Lange et al., 2008; Dunn et al., 2012; Smith et al., 2015; Brunius et al., 2016; Liu et al., 2020b). Including multiple independent and pooled PD1074 samples in each batch can mitigate these issues. A plasticizer contamination event precluded us from quantitatively assessing the performance of the IBAT controls in the LC-MS experiment.

### Quality control for NMR and LC-MS

Stable spectral features (LC-MS and NMR peak height) were rank transformed (add_group_rank.py, SECIMTools version 22.3.23, https://github.com/secimTools/SECIMTools). Atypical samples and potential feature artifacts were identified using the following SECIMTools QC tools (https://github.com/secimTools/SECIMTools): pairwise standard Euclidean distance (SED, standardized_euclidean_distance.py), principal component analysis (PCA, principle_component_analysis.py), sample density distributions (distribution_samples.py), coefficient of variation (CV, coefficient_variation_flags.py), and Bland-Altman plots (BA, bland_altman_plot.py).

BA plots on PD1074 pools and individual PD1074 samples within a batch were used to visualize feature alignment variation and identify successful alignment across batches. Per feature CV was used to identify aberrant features. Sample outliers and/or atypical samples were identified based on sample distribution, PCA, and SED plots. Samples whose distance in SED plots to other samples did not cross the 95% percentile for the distribution of pairwise distances were manually examined. (Supplementary Table S2). PD1074 LSCP sample “aos54” failed the QC assessment for NMR. The PD1074 LSCP samples “aos53” and “aos41” failed the QC assessment for RP LC-MS datasets. Test strain “aos49” in batch 5 was removed from all datasets, and test strain “aos25” in batch 1 was removed from the HILIC LC-MS positive dataset.

### Meta-analysis provides similar inferences to mixed models

Meta-analysis is a statistical analysis that combines summary statistics instead of an analysis of individual samples (Lin and Zeng, 2010; Liu, 2021). Batch effects across experiments are an enormous problem in metabolomics experiments, and the complications in adequately addressing this in mixed model analyses is a well-known problem (Liu et al., 2020b; Liu, 2021). In meta-analysis, an effect size is calculated for each study (in this case within each batch) and then combined and weighed by the individual study sample sizes (Hall and Rosenthal, 1995; Rosenthal and DiMatteo, 2001). Meta-analysis has been shown to be equivalent to more complex linear model approaches on individual data on larger sample sizes (Lin and Zeng, 2010). We demonstrate that in this experiment meta-analysis, even with relatively small sample sizes per group (n = 6 for test samples), is very similar to a mixed effects model with the variance modeled appropriately (Liu, 2021). A fixed-effect model is used here as there are only two batches per strain. We use the usual fixed-effect model effect size estimate (Hall and Rosenthal, 1995; Rosenthal and DiMatteo, 2001):

$${\bar{\theta }}_{w}=\frac{\overset{k}{\underset{i=1}{\sum }}w_{i}\theta_{i}}{\overset{k}{\underset{i=1}{\sum }}w_{i}}$$

where w_i_ is a weight calculated as the inverse of variance for the effect size in batch *i*, where i = 1 or 2, and ${\bar{\theta }}_{w}$ is the effect size of interest inferred from the individual effect sizes in batches. For the ANOVA comparison, the effect size is calculated as:

$$\bar{\theta }=\frac{{lsmean}_{test}-{lsmean}_{PD1074}}{sd}$$

$$sd=\sqrt{n}*se$$

To illustrate the comparability between these approaches, we compare the linear model by batch *l*, where strain *i* is the independent variable and ion signal for each spectral feature *m,* and test replicate *j* is the response variable*:*

$$Y_{mlij}=\mu +{{batch}_{l}+strain}_{i}+e_{lij}$$

The effect size estimates are consistent between the two approaches (e.g., Supplementary Figure S3).

### Meta-analysis for each test strain (meta-strain model)

All replicates of a test strain were contained within two sequential batches: however, different test strains within the same study span multiple sets. We used meta-analysis by feature to compare the test strain to the control, where each batch is treated as an ‘experiment’ using the fixed effects (FE) model and standardized mean difference (SMD) (Kirpich et al., 2018). We then calculated the strain effect relative to the control for each strain within each batch. Positive effect sizes indicate that the test strain had a higher peak than PD1074 for a given chemical feature. Similarly, negative effect sizes indicate that PD1074 had a higher peak than the test strain. Estimates of the strain effect were combined across batches using the meta-analytic weight. This approach does not require the batch effect to be estimated and adjusted-instead the strain effect relative to PD1074 is estimated within the batch.

### Meta-analysis to compare test strains (meta-study model)

We demonstrate how we can compare individual features across strains using meta-analysis with a statistical evaluation of the joint evidence of NMR polar feature 2.3291 ppm (Supplementary Figure S4). We wrapped the metaphor R package (Viechtbauer, 2010) in python (meta_analysis.py) to calculate batch-level summary statistics and model estimates. Here since there is a common reference, the strain effects can be compared directly using a meta-analysis. The python script has also been wrapped for implementation in Galaxy (https://galaxyproject.org/) and is available through SECIMTools version 22.3.24 or newer (https://github.com/secimTools/SECIMTools). The python code includes an option to output a forest plot for each feature.

### NMR spectra annotation

The 2D experiments HSQC, HSQC-TOCSY, and TOCSY were collected from a pooled sample composed of all individual PD1074 samples. Peak picking and spectral matching against publicly available databases (*i.e.*, HMDB (Wishart et al., 2022), BMRB (Ulrich et al., 2008), and NMRShiftDB (Kuhn and Schlorer, 2015)) were carried out by COLMARm (Complex Mixture Analysis by NMR) using 0.04 and 0.3 ppm chemical shift cutoffs for ^1^H and ^13^C respectively, and a matching ratio cutoff of 0.6. Annotated compounds where the feature with the highest effect size was selected for annotated compounds with more than one significant feature. After a list of compounds was identified, WormFlux (Yilmaz and Walhout, 2016) (version iCEL1314), a web based metabolic network modeling of *C. elegans* was used to visualize the putative pathways for CM mutants on the *C. elegans* metabolic network (Annotation confidence scores (Walejko et al., 2018); Supplementary Table S3).

### Elemental formula assignment LC-MS

SIRIUS (version 4.9.12), a java-based software framework for the analysis of LC-MS/MS data of metabolites and other small molecules (Dührkop et al., 2019), was used to generate elemental formulas. The mgf output from SLAW containing MS/MS information was used as input. For molecular formula identification, the default Orbitrap settings were used with an upper mass limit of 850 Da due to the high combinatorial possibilities and decrease in accuracy at high molecular weight. Only formulas found in databases were considered and all precursors were assumed to be ± 1 for positive and negative mode, respectively. SIRIUS ranks molecular formulas for each compound individually using accurate mass, fragmentation trees, and isotope pattern analysis (Dührkop et al., 2019). ZODIAC takes the fragmentation trees as input and re-ranks the molecular formula candidates by taking similarities of compounds in the dataset into account (Ludwig et al., 2019). ZODIAC (Ludwig et al., 2019) and SIRIUS (Dührkop et al., 2019) scores are available at https://github.com/artedison/metaanalysis/tree/main/LC-MS/SIRIUS_output (Supplementary Table S4).

## Results

We developed a method to identify stable spectral features and identify differences in features between test strains and controls and among test strains using a straightforward meta-analytic approach. We collected 104 test samples for three genetic studies across six batches to produce five analytical datasets from two complementary technologies commonly used in untargeted metabolomics (3 LC-MS and 2 NMR; Figure 1, 2, and Supplementary Figure S2).

Stringent quality assurance/quality controls (QA/QC) combined with a focus on spectral features consistently detected in PD1074 identified: 3,953 stable spectral features in reversed phase (RP) LC-MS positive, 377 in RP LC-MS negative, 199 in hydrophilic interaction liquid chromatography (HILIC) LC-MS positive, 585 in NMR polar, and 487 in NMR non-polar datasets.

### Meta-analysis (meta-strain model) identifies differences in spectral features between test and reference strains without the need for complex normalization

For each spectral feature, the difference between the PD1074 individual LSCP (n = 6–10) and the test strain (n = 2–6) was estimated within the batch. Here, the batch effect is not estimated. Instead, we identified statistically significant spectral features by performing a meta-analysis across batches for each test strain (Hedges and Olkin, 1985) (Figure 2E). Inferences are similar to a more complex mixed model approach (Supplementary Figure S3), as predicted by studies that have compared individual analyses and meta-analytic approaches for larger sample sizes (Liu, 2021). An advantage of the meta-analysis is the ability to apply this technique generally, especially when there may be complex patterns of variance across batches such as those present in large cohort studies and/or due to technical variation (*e.g.*, after an instrument interruption). This is because the experimental design allows the estimation of the treatment effect within each batch.

**FIGURE 3:**
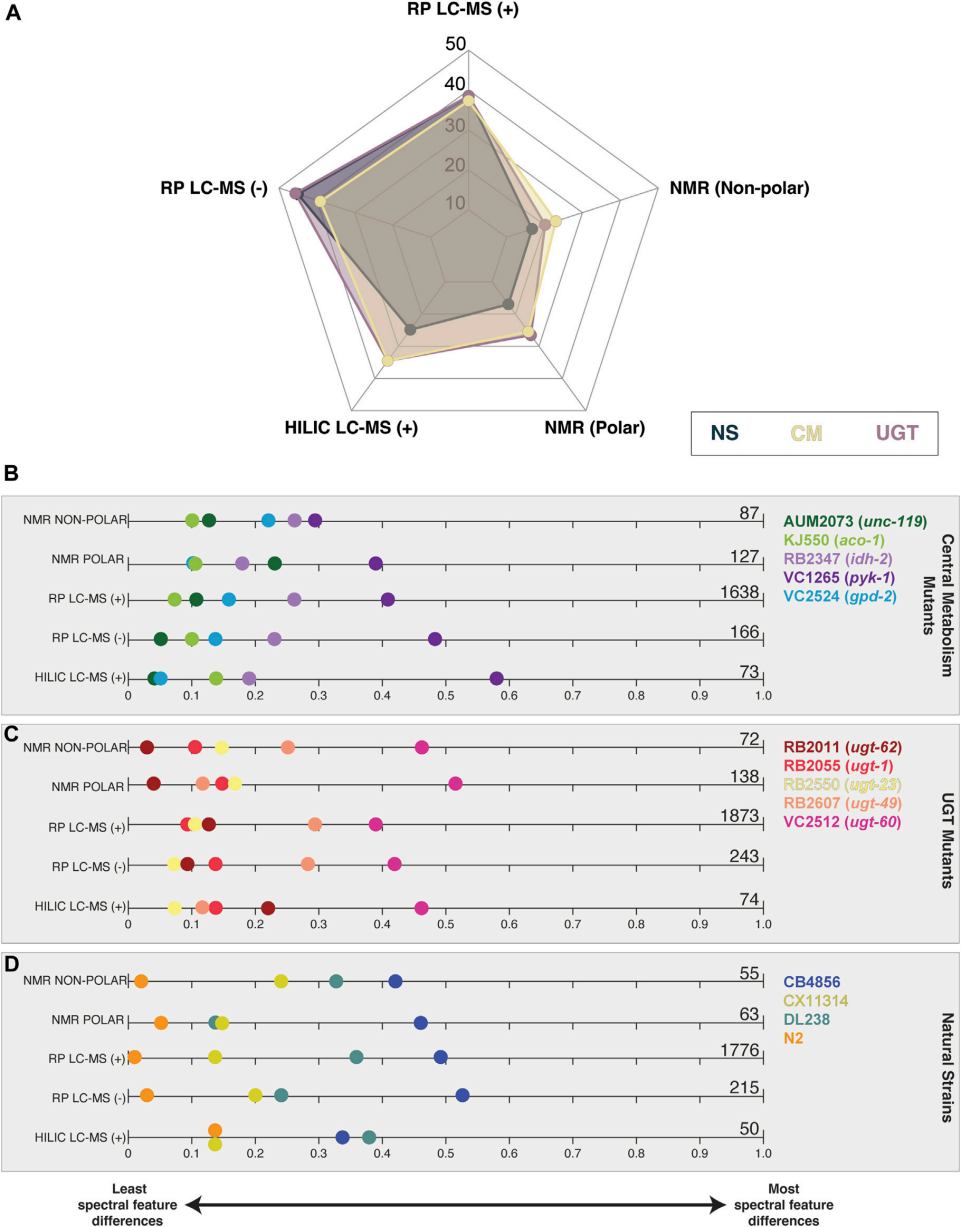
Summary of significant spectral features found in each analytical platform and across the three studies. **(A)** Percent of significant features. The total number of significant features in all strains, by study, is used as the denominator for each of the five technologies. Significant spectral features identified in at least one strain by study are displayed for **(B)** central metabolism mutants, **(C)** UGT mutants, and **(D)** natural strains. Zero indicates the strain has no significant spectral feature differences from PD1074, while one indicates that all spectral feature differences from PD1074 are present in that strain. Significant feature totals are summarized at the end of the plot and detailed in Table 1.

We calculated the number of significant spectral features for each test strain compared to PD1074 (Table 1). We see a similar pattern across platforms for the percentage of significant features identified across the three studies, with the highest percentage found in the RP LC-MS (-) dataset (Figure 3A). The highest percentage of significant spectral features was 58% in the CM mutation study. In the individual strains, the CM mutant, VC1265 (*pyk-1*) had the largest overall effect across platforms and fractions, followed by RB2347 (*idh-2*). AUM2073 (*unc-119*) and KJ550 (*aco-1*) had the smallest overall effects (Figure 3B). For the UGT mutants, VC2512 (*ugt-60*) had the largest overall effect, followed by RB2607 (*ugt-49*). RB2011 (*ugt-62*) had the smallest overall effect (Figure 3C and Supplementary Figure S5). In the NS, the most genetically divergent strains from PD1074 (CB4856 and DL238) had the largest overall effect in both platforms, and N2 had a small set of differences, as expected, since PD1074 has minor genetic differences with N2 (SI, Figure 3D and Supplementary Figure S6). In the LC-MS datasets we were able to determine elemental formulas for 26.6%, 56%, and 83.4% of the stable spectral features in RP LC-MS (+), RP LC-MS (-), and HILIC LC-MS (+), respectively. Elemental formulas for significant features ranged between 27.9% and 79.1% (Table 1).

**TABLE 1:** Summary of significant spectral features found in all three studies across NMR and LC-MS. The total number of significant spectral features (*p* < 0.05) for a given strain and each analytical platform are listed. SIRIUS (Dührkop et al., 2019) was used to determine elemental formulas for all MS/MS features.

Study Group	Strain	Number of significant spectral features				
		NMR		LC-MS		
		Non-polar	Polar	RP+	RP-	HILIC
Central metabolism mutans (CM)	AUM2073	11	29	175	8	3
	KJ550	9	14	110	17	10
	RB2347	23	22	421	38	14
	VC1265	25	49	671	79	42
	VC2524	19	13	261	24	4
	Total CM sig. features by platform	87	127	1638	166	73
UGT mutants (UGT)	RB2011	2	6	237	21	16
	RB2055	8	20	161	34	10
	RB2550	11	23	200	18	5
	RB2607	18	17	539	69	9
	VC2512	33	72	736	101	34
	Total UGT sig. features by platform	72	138	1873	243	74
Natural Strains (NS)	N2	1	3	22	6	7
	DL238	18	15	631	52	9
	CX11314	13	16	254	44	7
	CB4856	23	29	869	113	17
	Total NS sig. features by platform	55	63	1776	215	50
	Total sig. feautres within platform	146	228	2541	281	115
Total number of sig. features with elemental formulas				709	152	91

### Identification of spectral features significant in mutant and natural strains are of interest for follow-up compound identification

**FIGURE 4:**
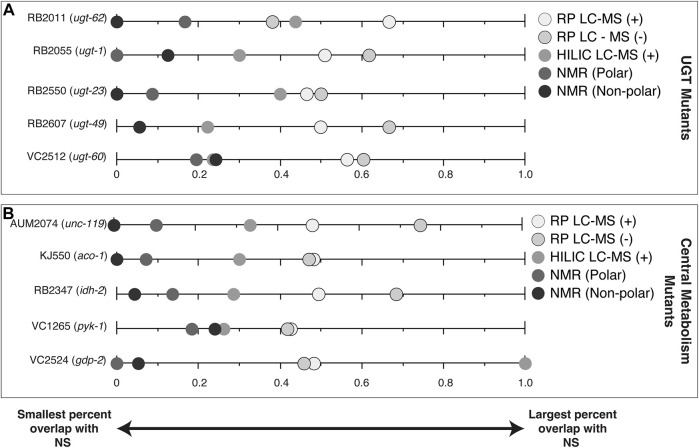
Percent of significant features for each of the mutant studies (CM and UGT) that are also significant in at least one NS by analytical platform. **(A)** UGT mutants **(B)** CM mutants. Data points at zero indicate the analytical platform detected no significant spectral features shared between the mutant strain and a natural strain. Data points at one indicate all significant spectral features for the mutant strain are shared with a natural strain for that analytical platform.

One way of reducing the number of features for follow-up is to examine features that are affected by a gene knock-out that also vary in nature. The percentage of significant features in each of the mutant studies (CM and UGT) that overlapped in at least one NS (Figure 4, Supplementary Figure S7, S8) are features of interest for compound identification. CM mutant strains VC2524 (*gdp-2*), AUM2073 (*unc-119*) and RB2347 (*idh-2*) share 100%, 75% and 68% of their significant features with a NS, respectively. UGT mutants, RB2607 (*ugt-49*) and RB2055 (*ugt-1*) share 67% and 62% of their significant features with a NS, respectively.

We focused on compounds affected in any CM mutants and used those to identify which UGTs and NSs had genetic variation in those same compounds for the NMR polar data. Using COLMAR (Zhang et al., 2009), we identified three putative compounds significant in strains from all three studies. Of the 35 putative compounds showing evidence for metabolic variation in the NMR data, 13 were annotated (Supplementary Table S3).

**FIGURE 5:**
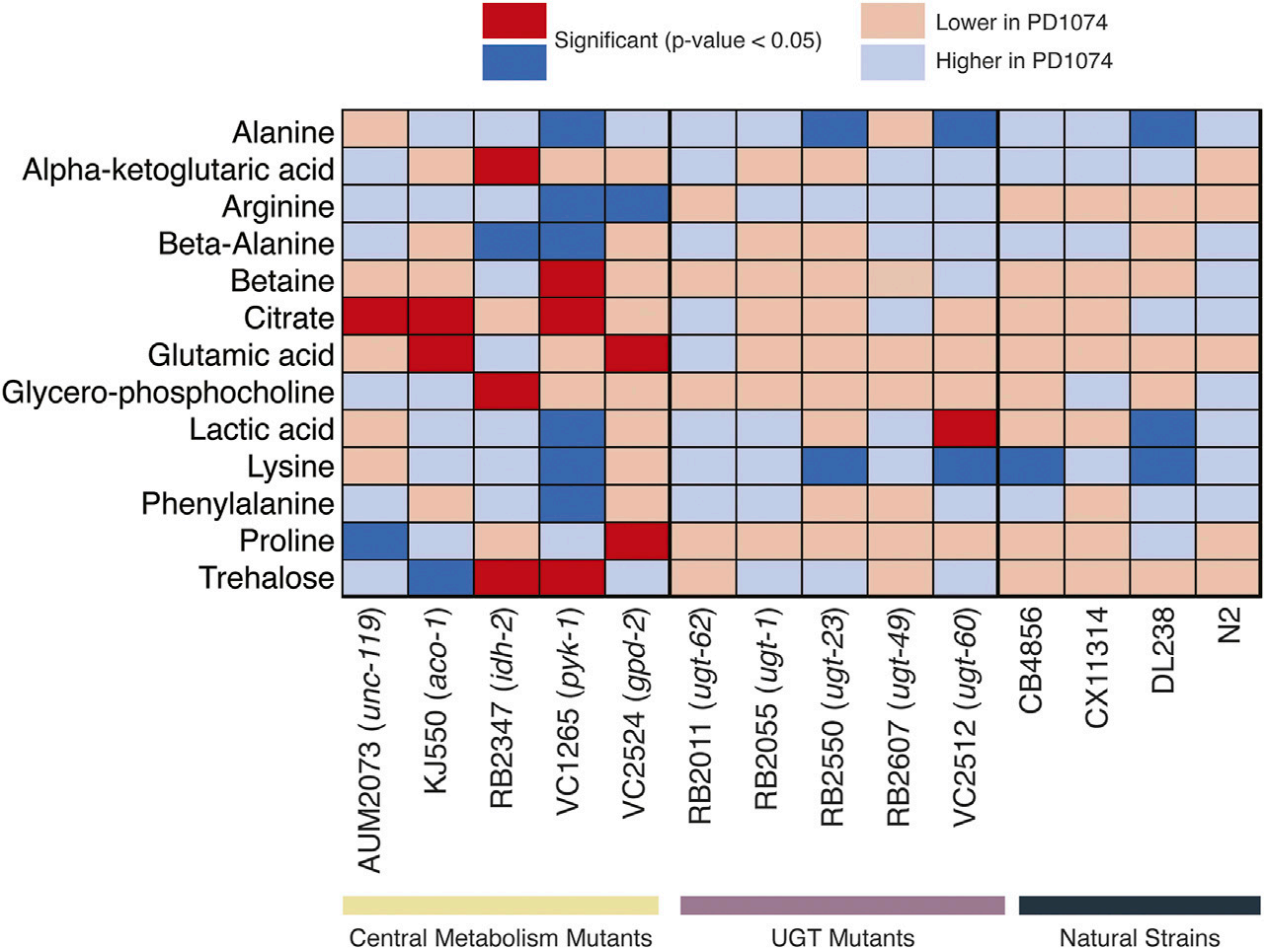
Heatmap of metabolites identified by NMR. Significant NMR spectral features in the central metabolism mutants are compared across UGT mutants and natural strains. Deep blue boxes indicate the metabolite is significant and more abundant in PD1074 compared to the test strain. Deep red boxes indicate the metabolite is significant and more abundant in the test strain. Light colored boxes indicate the direction of effect when the metabolite is not significantly different between PD1074 and the test strain. For compounds with more than one significant feature, the highest effect size feature is used for this figure. The significant compound list provides metabolites to pursue in subsequent experiments (Supplementary Table S3).

Nine putative compounds show metabolic variation in response to the *pyk-1* mutation (Figure 5). The mutation in *pyk-1* affects a large portion of the metabolome. The gene *pyk-1*, is involved in one of the last enzymes of glycolysis, encoding for pyruvate kinase and responsible for glycolytic ATP production. The depletion of lactic acid production is consistent with the mutation in *pyk-1* (Luz et al., 2016) in the strain VC1265. We saw the depletion of lactic acid in DL238 (NS), and an increase in VC2512 (*ugt-60*) (Figure 5). As expected, none of the 13 compounds identified in the NMR polar dataset were significant in N2 (Figure 5). Interestingly, annotated compounds were also similar to PD1074 in CX11314 (NS), RB2055 (*ugt-1*), RB2607 (*ugt-49*), and RB2011 (*ugt-62*).

#### Meta-analysis (meta-study model) identifies differences in spectral features between test strains across independent batches

**FIGURE 6:**
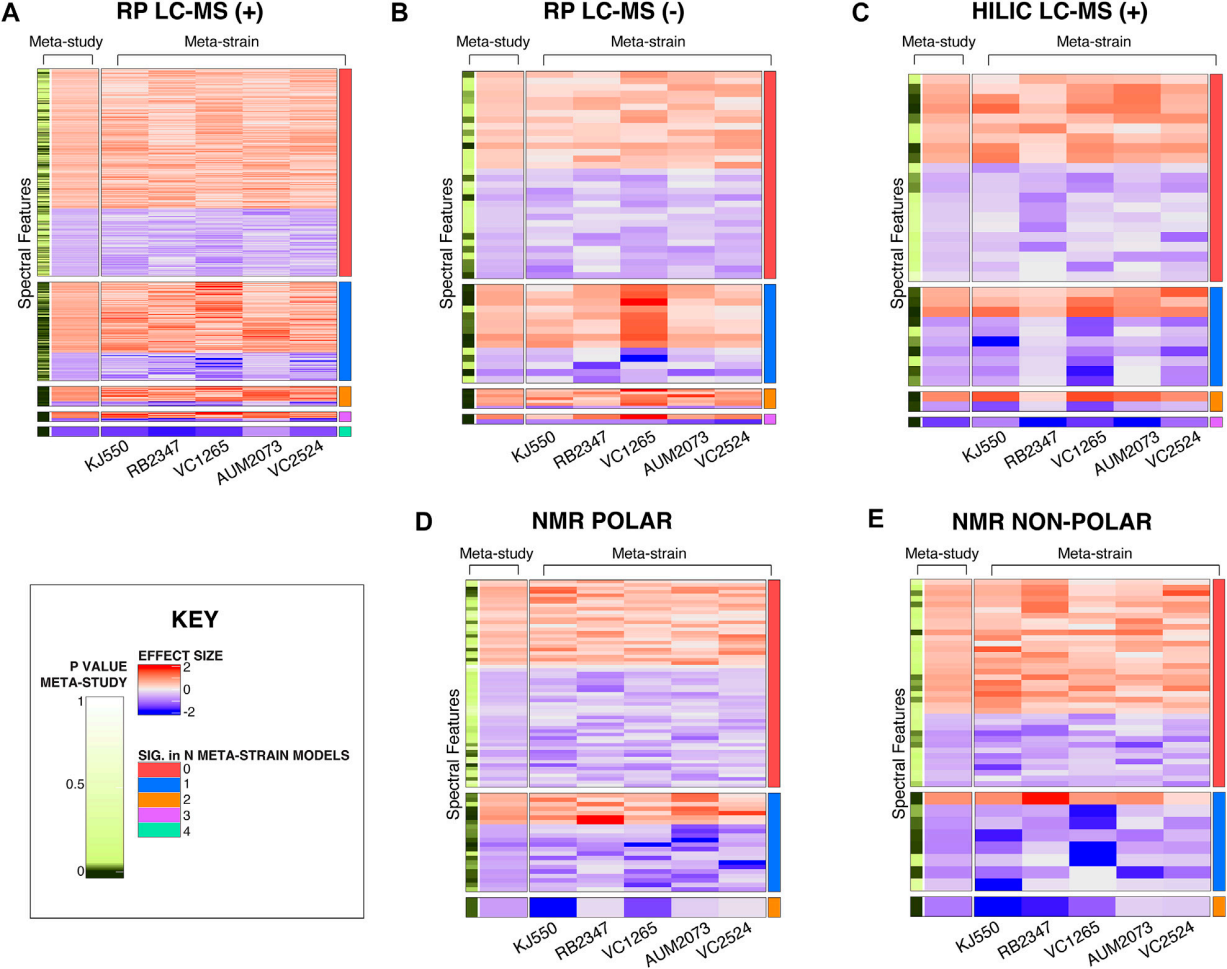
Heatmaps of significant spectral features in the CM mutant study. **(A)** RP LC-MS positive mode **(B)** RP LC-MS negative mode **(C)** HILIC LC-MS positive mode **(D)** NMR polar and **(E)** NMR non-polar. The first two columns pertain to the meta-study results. The left-most column indicates the significant features found in the meta-study model, followed by the estimated effect size in the meta-study model across CM mutants. The following five columns are the effect sizes for individual strains, and the contents of the cell are the estimates of the effect of that strain (column), for that feature (row) compared to PD1074 from the meta-strain model. Features where the effect sizes are consistent for all strains are included. The effect sizes range from (2 to -2). Positive effect sizes (*i.e.*, the strain had a higher peak at that given metabolic feature than PD1074) are displayed in red. Negative effect sizes (*i.e.*, PD1074 had a higher peak at that given metabolic feature than the test strain) are displayed in blue. The right-most column indicates the number of strains in which a given spectral feature is statistically significant.

In addition to the more standard approach where differences between a single test strain and their respective control strain are compared, the meta-analysis (meta-study model) identifies differences in spectral features between test strains across batches. Heatmaps of the CM mutant study in Figure 6 demonstrate the unique benefits of this approach. Each heatmap shows features where the effect sizes are in the same direction for the CM mutant study (Supplementary Figure S5, S6 for UGT and NS results). For the meta-study model of the CM mutants, each feature is compared across the five strains shown in Figure 6. In the NS, N2 was excluded from meta-study comparisons due to the genetic similarity of N2 to the control strain PD1074 (Figure 3, Supplementary Figure S8). We calculated the number of features that were not significant in any of the individual meta-strain comparisons but are significant when compared *via* the meta-study (Supplementary Table S6). To visualize the effect size of a specific feature across strains within the meta-study model and to contextualize features within a study group we visualized features significant in the meta-study model using a forest plot. An example of one such comparison can be seen across the NS in Supplementary Figure S4 **.**

## Discussion

Modern analytical technologies can detect many more spectral features than can be identified and interpreted. While there are many gains to be made by uncovering the richness of the larger metabolome, here we take a narrower approach by focusing only on features that we can detect consistently in the strain PD1074. This choice results in fewer features but enables us to compare these features over time across a variety of studies. However, the principle behind this approach is not limited to a single strain/genotype but is constrained by the size of the extraction batch. If multiple strains are present across batches, stable features can be identified from any strain. In studies of non-model systems including multiple ‘control’ samples in each batch can be used to estimate the treatment effect for each group relative to the control within each batch. The treatment effect for the study is found using the meta-analytic approach of a weighted average across batches. Further, if all groups in the study are present in each batch, there is no need to prioritize feature selection for any one group, instead, spectral features consistently present within any of the groups can be prioritized for future studies so that database matching and, ultimately, compound identification efforts are focused on the most likely biologically important spectral features. This aspect is important as studies increase in size and complexity (Annesley, 2003; Lewis et al., 2008; Gebauer et al., 2016; Wehrens et al., 2016; Menni et al., 2017; Chamberlain et al., 2019; Gouveia et al., 2021).

Recent computational advances now enable joint alignment and feature selection when batches are augmented with a common QC standard like a BRM included (Sousa and Ferreira, 2013). The IBAT control used here is a BRM. BRM are important tools for understanding batch variation, but do not allow for the assessment of feature stability due to individual variation among biological replicates. The study design proposed leverages biological variation within batches to obviate the need for estimating and correcting for the batch effect.

The inclusion of QC samples is critical in large-scale studies (Peng et al., 2014; Han and Li, 2020; Gouveia et al., 2021). Our inclusion of individual PD1074 biological replicates and PD1074 pools during sample generation, analytical measurement, and data processing is an extra layer of replication. We used the biological replicates of PD1074 samples both for the selection of stable spectral peaks, to enable the estimation of strain effects within batches; and to provide a reference strain for combining data across studies. This conservative approach focused the experiment, and our attention on spectral features likely to be present in a subsequent independently prepared MS2 experiment in the compound identification process, and not on spectral features present sporadically due to variation in growth or extraction.

Using this approach, we annotated 13 compounds in our NMR polar dataset. For the confident identification of features, compound annotation using an orthogonal method, such as LC-MS, is required. Compound identification approaches for LC-MS require additional experimentation for confident annotations. As a first step in the compound identification pipeline, we calculated elemental formulas based on accurate mass (within 5 ppm) using SIRIUS (Dührkop et al., 2019). A set of stable features simplifies MS/MS experiments by enabling the scientist to target spectral features of biological interest (*e.g.*, different in abundance in multiple experiments). Focusing on spectral features that are consistently detectable enables the investigator to predict the presence of those features in future samples rather than relying on the potential intersection among MS/MS features collected by data-dependent acquisition (DDA) or iterative DDA approaches. ^1^H and ^13^C 1D NMR, collisional cross-section (CCS), *in silico* prediction, retention time prediction, and MS/MS fragmentation data collection can be prioritized for target features identified with this approach (Bouwmeester et al., 2019; Bonini et al., 2020; Soper-Hopper et al., 2020; Borges et al., 2021; Das et al., 2022). *In silico* prediction methods for NMR and MS/MS have improved accuracy, although ambiguity is expected to remain, especially for large molecular weight compounds (Das et al., 2020). However, even with MS/MS data, compounds may elude identification.

Once compounds are identified, network modeling and pathway mapping can aid in understanding the relationship between metabolites within and between pathways. However, mapping metabolites in pathways is complicated because many metabolites are involved in multiple pathways and/or pathways are incomplete. The genetic mutation approach used to annotate gene function in pathways has had limited success in untargeted metabolomics because of the scope of the experiments and the necessity of multiple subsequent experiments to discern pathway-gene relationships. With large numbers of unknown spectral features, this problem is complex. Meta-analysis allows for the identification of significant spectral features in a straightforward manner when batch effects complicate mixed effects models. For forward and reverse genetic mutation studies, the meta-study model serves as a valuable approach where effect sizes can be calculated and used to assess patterns across an entire pathway. Similarly, untargeted studies of collections of genotypes (Noble et al., 2021) using a reference genotype, in this case PD1074, can leverage data across experiments and increase the utility of untargeted metabolomics for genetic studies (Blazenovic et al., 2018). Meta-analysis can be used to make formal statistical comparisons across studies conducted over long periods of time or in different labs without the need to estimate and correct for batch effects. (Yoshimura et al., 2019).

## Data Availability

The datasets presented in this study can be found in online repositories. The names of the repository/repositories and accession number(s) can be found in the article/Supplementary Material.

## References

[B1] Annesley T. M. (2003). Ion suppression in mass spectrometry. Clin. Chem. 49 (7), 1041–1044. 10.1373/49.7.1041 12816898

[B2] Barupal D. K. Fan S. Wancewicz B. Cajka T. Sa M. Showalter M. R. (2018). Generation and quality control of lipidomics data for the alzheimer's disease neuroimaging initiative cohort. Sci. Data 5, 180263. 10.1038/sdata.2018.263 30457571 PMC6244184

[B3] Beisken S. Eiden M. Salek R. M. (2015). Getting the right answers: Understanding metabolomics challenges. Expert Rev. Mol. diagn. 15 (1), 97–109. 10.1586/14737159.2015.974562 25354566

[B4] Blazenovic I. Kind T. Ji J. Fiehn O. (2018). Software tools and approaches for compound identification of LC-MS/MS data in metabolomics. Metabolites 8 (2), E31. 10.3390/metabo8020031 PMC6027441 29748461

[B5] Bonini P. Kind T. Tsugawa H. Barupal D. K. Fiehn O. (2020). Retip: Retention time prediction for compound annotation in untargeted metabolomics. Anal. Chem. 92 (11), 7515–7522. 10.1021/acs.analchem.9b05765 32390414 PMC8715951

[B6] Borges R. M. Colby S. M. Das S. Edison A. S. Fiehn O. Kind T. (2021). Quantum chemistry calculations for metabolomics. Chem. Rev. 121 (10), 5633–5670. 10.1021/acs.chemrev.0c00901 33979149 PMC8161423

[B7] Bouwmeester R. Martens L. Degroeve S. (2019). Comprehensive and empirical evaluation of machine learning algorithms for small molecule LC etention time prediction. Anal. Chem. 91 (5), 3694–3703. 10.1021/acs.analchem.8b05820 30702864

[B8] Broadhurst D. Goodacre R. Reinke S. N. Kuligowski J. Wilson I. D. Lewis M. R. (2018). Guidelines and considerations for the use of system suitability and quality control samples in mass spectrometry assays applied in untargeted clinical metabolomic studies. Metabolomics 14 (6), 72. 10.1007/s11306-018-1367-3 29805336 PMC5960010

[B9] Brunius C. Shi L. Landberg R. (2016). Large-scale untargeted LC-MS metabolomics data correction using between-batch feature alignment and cluster-based within-batch signal intensity drift correction. Metabolomics 12 (11), 173. 10.1007/s11306-016-1124-4 27746707 PMC5031781

[B10] Burgess D. J. (2021). The TOPMed genomic resource for human health. Nat. Rev. Genet. 22 (4), 200. 10.1038/s41576-021-00343-x 33654294

[B11] Cajka T. Fiehn O. (2016). Toward merging untargeted and targeted methods in mass spectrometry-based metabolomics and lipidomics. Anal. Chem. 88 (1), 524–545. 10.1021/acs.analchem.5b04491 26637011

[B12] *C. elegans* Sequencing Consortium (1998). Genome sequence of the nematode *C. elegans*: A platform for investigating biology. Science 282 (5396), 2012–2018. 10.1126/science.282.5396.2012 9851916

[B13] Chamberlain C. A. Rubio V. Y. Garrett T. J. (2019). Impact of matrix effects and ionization efficiency in non-quantitative untargeted metabolomics. Metabolomics 15 (10), 135. 10.1007/s11306-019-1597-z 31584114

[B14] Chambers M. C. Maclean B. Burke R. Amodei D. Ruderman D. L. Neumann S. (2012). A cross-platform toolkit for mass spectrometry and proteomics. Nat. Biotechnol. 30 (10), 918–920. 10.1038/nbt.2377 23051804 PMC3471674

[B15] Cook D. E. Zdraljevic S. Roberts J. P. Andersen E. C. (2017). CeNDR, the *Caenorhabditis elegans* natural diversity resource. Nucleic Acids Res. 45 (D1), D650–D7. 10.1093/nar/gkw893 27701074 PMC5210618

[B16] Das S. Edison A. S. Merz K. M. Jr (2020). Metabolite structure assignment using in silico NMR techniques. Anal. Chem. 92 (15), 10412–10419. 10.1021/acs.analchem.0c00768 32608974 PMC8045457

[B17] Das S. Tanemura K. A. Dinpazhoh L. Keng M. Schumm C. Leahy L. (2022). *In silico* collision cross section calculations to aid metabolite annotation. J. Am. Soc. Mass Spectrom. 33, 750–759. 10.1021/jasms.1c00315 35378036 PMC9277703

[B18] De Livera A. M. Sysi-Aho M. Jacob L. Gagnon-Bartsch J. A. Castillo S. Simpson J. A. (2015). Statistical methods for handling unwanted variation in metabolomics data. Anal. Chem. 87 (7), 3606–3615. 10.1021/ac502439y 25692814 PMC4544854

[B19] Delaglio F. Grzesiek S. Vuister G. W. Zhu G. Pfeifer J. Bax A. (1995). NMRPipe: A multidimensional spectral processing system based on UNIX pipes. J. Biomol. NMR 6 (3), 277–293. 10.1007/BF00197809 8520220

[B20] Dührkop K. Fleischauer M. Ludwig M. Aksenov A. A. Melnik A. V. Meusel M. (2019). Sirius 4: A rapid tool for turning tandem mass spectra into metabolite structure information. Nat. Methods 16 (4), 299–302. 10.1038/s41592-019-0344-8 30886413

[B21] Dunn W. B. Wilson I. D. Nicholls A. W. Broadhurst D. (2012). The importance of experimental design and QC samples in large-scale and MS-driven untargeted metabolomic studies of humans. Bioanalysis 4 (18), 2249–2264. 10.4155/bio.12.204 23046267

[B22] Edison A. S. Hall R. D. Junot C. Karp P. D. Kurland I. J. Mistrik R. (2016). The time is right to focus on model organism metabolomes. Metabolites 6 (1), E8. 10.3390/metabo6010008 PMC4812337 26891337

[B23] Fan S. Kind T. Cajka T. Hazen S. L. Tang W. H. W. Kaddurah-Daouk R. (2019). Systematic error removal using random forest for normalizing large-scale untargeted lipidomics data. Anal. Chem. 91 (5), 3590–3596. 10.1021/acs.analchem.8b05592 30758187 PMC9652764

[B24] Fang C. Luo J. (2019). Metabolic GWAS-based dissection of genetic bases underlying the diversity of plant metabolism. Plant J. 97 (1), 91–100. 10.1111/tpj.14097 30231195

[B25] Federer W. T. Reynolds M. Crossa J. (2001). Combining results from augmented designs over sites. Agron. J. 93 (93), 389–395. 10.2134/agronj2001.932389x

[B26] Federer W. T. Zelen M. (1966). Analysis of multifactor classifications with unequal numbers of observations. Biometrics 22 (3), 525–552. 10.2307/2528186 5970554

[B27] Federer WtaS C. S. Schlottfeldt C. S. (1954). The use of covariance to control gradients in experiments. Biometrics 10 (2), 282–290. 10.2307/3001881

[B28] Fiehn O. Wohlgemuth G. Scholz M. Kind T. Lee D. Y. Lu Y. (2008). Quality control for plant metabolomics: Reporting MSI-compliant studies. Plant J. 53 (4), 691–704. 10.1111/j.1365-313X.2007.03387.x 18269577

[B29] Gebauer J. Gentsch C. Mansfeld J. Schmeisser K. Waschina S. Brandes S. (2016). A genome-scale database and reconstruction of *Caenorhabditis elegans* metabolism. Cell. Syst. 2 (5), 312–322. 10.1016/j.cels.2016.04.017 27211858

[B30] Girard L. R. Fiedler T. J. Harris T. W. Carvalho F. Antoshechkin I. Han M. (2007). WormBook: The online review of *Caenorhabditis elegans* biology. Nucleic Acids Res. 35, D472–D475. 10.1093/nar/gkl894 17099225 PMC1669767

[B31] Gouveia G. J. Shaver A. O. Garcia B. M. Morse A. M. Andersen E. C. Edison A. S. (2021). Long-Term metabolomics reference material. Anal. Chem. 93 (26), 9193–9199. 10.1021/acs.analchem.1c01294 34156835 PMC8996483

[B32] Hall J. A. Rosenthal R. (1995). Interpreting and evaluating meta-analysis. Eval. Health Prof. 18 (4), 393–407. 10.1177/016327879501800404 10153164

[B33] Han W. Li L. (2020). Evaluating and minimizing batch effects in metabolomics. Mass Spectrom. Rev. 41, 421–442. 10.1002/mas.21672 33238061

[B34] Hasegawa K. Miwa S. Tsutsumiuchi K. Miwa J. (2010). Allyl isothiocyanate that induces GST and UGT expression confers oxidative stress resistance on *C. elegans*, as demonstrated by nematode biosensor. PLoS One 5 (2), e9267. 10.1371/journal.pone.0009267 20174640 PMC2822842

[B35] Hastings J. Mains A. Virk B. Rodriguez N. Murdoch S. Pearce J. (2019). Multi-Omics and genome-scale modeling reveal a metabolic shift during *C. elegans* aging. Front. Mol. Biosci. 6, 2. 10.3389/fmolb.2019.00002 30788345 PMC6372924

[B36] Hedges L. V. Olkin I. (1985). Statistical methods for meta-analysis. Orlando: Academic Press.

[B37] Helf M. J. Fox B. W. Artyukhin A. B. Zhang Y. K. Schroeder F. C. (2022). Comparative metabolomics with Metaboseek reveals functions of a conserved fat metabolism pathway in *C. elegans* . Nat. Commun. 13 (1), 782. 10.1038/s41467-022-28391-9 35145075 PMC8831614

[B38] Hodgkin J. (2001). What does a worm want with 20, 000 genes? Genome Biol. 2 (11), COMMENT2008. 10.1186/gb-2001-2-11-comment2008 11737938 PMC138976

[B39] Huaxu Yu T. H. Huan T. (2022). Comprehensive assessment of the diminished statistical power caused by nonlinear electrospray ionization responses in mass spectrometry-based metabolomics. Anal. Chim. Acta 1200, 339614. 10.1016/j.aca.2022.339614 35256134

[B40] Jones D. P. Park Y. Ziegler T. R. (2012). Nutritional metabolomics: Progress in addressing complexity in diet and health. Annu. Rev. Nutr. 32, 183–202. 10.1146/annurev-nutr-072610-145159 22540256 PMC4031100

[B41] Kenar E. Franken H. Forcisi S. Wörmann K. Häring H-U. Lehmann R. (2014). Automated label-free quantification of metabolites from liquid chromatography-mass spectrometry data. Mol. Cell. Proteomics 13 (1), 348–359. 10.1074/mcp.M113.031278 24176773 PMC3879626

[B42] Kim T. Tang O. Vernon S. T. Kott K. A. Koay Y. C. Park J. (2021). A hierarchical approach to removal of unwanted variation for large-scale metabolomics data. Nat. Commun. 12 (1), 4992. 10.1038/s41467-021-25210-5 34404777 PMC8371158

[B43] Kirpich A. S. Ibarra M. Moskalenko O. Fear J. M. Gerken J. Mi X. (2018). SECIMTools: A suite of metabolomics data analysis tools. BMC Bioinforma. 19 (1), 151. 10.1186/s12859-018-2134-1 PMC5910624 29678131

[B44] Kuhn S. Schlorer N. E. (2015). Facilitating quality control for spectra assignments of small organic molecules: nmrshiftdb2--a free in-house NMR database with integrated LIMS for academic service laboratories. Magn. Reson. Chem. 53 (8), 582–589. 10.1002/mrc.4263 25998807

[B45] Lange E. Tautenhahn R. Neumann S. Gropl C. (2008). Critical assessment of alignment procedures for LC-MS proteomics and metabolomics measurements. BMC Bioinforma. 9, 375. 10.1186/1471-2105-9-375 PMC2570366 18793413

[B46] Lewis G. D. Asnani A. Gerszten R. E. (2008). Application of metabolomics to cardiovascular biomarker and pathway discovery. J. Am. Coll. Cardiol. 52 (2), 117–123. 10.1016/j.jacc.2008.03.043 18598890 PMC3204897

[B47] Lin D. Y. Zeng D. (2010). On the relative efficiency of using summary statistics versus individual-level data in meta-analysis. Biometrika 97 (2), 321–332. 10.1093/biomet/asq006 23049122 PMC3412575

[B48] Liu K. H. Nellis M. Uppal K. Ma C. Tran V. Liang Y. (2020). Reference standardization for quantification and harmonization of large-scale metabolomics. Anal. Chem. 92 (13), 8836–8844. 10.1021/acs.analchem.0c00338 32490663 PMC7887762

[B49] Liu Q. Walker D. Uppal K. Liu Z. Ma C. Tran V. (2020). Addressing the batch effect issue for LC/MS metabolomics data in data preprocessing. Sci. Rep. 10 (1), 13856. 10.1038/s41598-020-70850-0 32807888 PMC7431853

[B50] Liu X. Locasale J. W. (2017). Metabolomics: A primer. Trends biochem. Sci. 42 (4), 274–284. 10.1016/j.tibs.2017.01.004 28196646 PMC5376220

[B51] Liu Z. (2021). Batch effect corrections in untargeted metabolomics. Gainesville, FL: University of Florida.

[B52] Ludwig M. Nothias L-F. Dührkop K. Koester I. Fleischauer M. Hoffmann M. A. (2019). Zodiac: Database-independent molecular formula annotation using gibbs sampling reveals unknown small molecules. bioRxiv, 842740.

[B53] Luz A. L. Godebo T. R. Bhatt D. P. Ilkayeva O. R. Maurer L. L. Hirschey M. D. (2016). From the cover: Arsenite uncouples mitochondrial respiration and induces a warburg-like effect in *Caenorhabditis elegans* . Toxicol. Sci. 152 (2), 349–362. 10.1093/toxsci/kfw093 27208080 PMC4960910

[B54] Marquez J. Flores J. Kim A. H. Nyamaa B. Nguyen A. T. T. Park N. (2019). Rescue of TCA cycle dysfunction for cancer therapy. J. Clin. Med. 8 (12), E2161. 10.3390/jcm8122161 PMC6947145 31817761

[B55] Martinez-Reyes I. Chandel N. S. (2020). Mitochondrial TCA cycle metabolites control physiology and disease. Nat. Commun. 11 (1), 102. 10.1038/s41467-019-13668-3 31900386 PMC6941980

[B56] MATLAB (2019). MATLAB and statistics toolbox release. Massachusetts, United States: The MathWorks, Inc.

[B57] Meech R. Hu D. G. McKinnon R. A. Mubarokah S. N. Haines A. Z. Nair P. C. (2019). The UDP-glycosyltransferase (UGT) superfamily: New members, new functions, and novel paradigms. Physiol. Rev. 99 (2), 1153–1222. 10.1152/physrev.00058.2017 30724669

[B58] Menni C. Zierer J. Valdes A. M. Spector T. D. (2017). Mixing omics: Combining genetics and metabolomics to study rheumatic diseases. Nat. Rev. Rheumatol. 13 (3), 174–181. 10.1038/nrrheum.2017.5 28148918

[B59] Misra B. B. (2020). Data normalization strategies in metabolomics: Current challenges, approaches, and tools. Eur. J. Mass Spectrom. 26 (3), 165–174. 10.1177/1469066720918446 32276547

[B60] Molon M. Dampc J. Kula-Maximenko M. Zebrowski J. Molon A. Dobler R. (2020). Effects of temperature on lifespan of *Drosophila melanogaster* from different genetic backgrounds: Links between metabolic rate and longevity. Insects 11 (8), E470. 10.3390/insects11080470 PMC7469197 32722420

[B61] Myers O. D. Sumner S. J. Li S. Barnes S. Du X. A-O. (2017). One step forward for reducing false positive and false negative compound identifications from mass spectrometry metabolomics data: New algorithms for constructing extracted ion chromatograms and detecting chromatographic peaks. Anal. Chem. 89, 1520–6882. 10.1021/acs.analchem.7b00947 28752754

[B62] Noble L. M. Rockman M. V. Teotonio H. (2021). Gene-level quantitative trait mapping in *Caenorhabditis elegans* . G3 (Bethesda) 11 (2), jkaa061. 10.1093/g3journal/jkaa061 33693602 PMC8022935

[B63] Patterson R. E. Kirpich A. S. Koelmel J. P. Kalavalapalli S. Morse A. M. Cusi K. (2017). Improved experimental data processing for UHPLC–HRMS/MS lipidomics applied to nonalcoholic fatty liver disease. Metabolomics 13 (142), 142. 10.1007/s11306-017-1280-1

[B64] Peng B. Li H. Peng X. X. (2015). Functional metabolomics: From biomarker discovery to metabolome reprogramming. Protein Cell. 6 (9), 628–637. 10.1007/s13238-015-0185-x 26135925 PMC4537470

[B65] Peng J. Chen Y. T. Chen C. L. Li L. (2014). Development of a universal metabolome-standard method for long-term LC-MS metabolome profiling and its application for bladder cancer urine-metabolite-biomarker discovery. Anal. Chem. 86 (13), 6540–6547. 10.1021/ac5011684 24877652

[B66] Pluskal T. CastilloFau - Villar-Briones S. A. Villar-Briones A Fau - Oresic M. Oresic M. (2010). MZmine 2: Modular framework for processing, visualizing, and analyzing mass spectrometry-based molecular profile data. BMC Bioinforma. 395, 1471–2105. 10.1186/1471-2105-11-395 PMC2918584 20650010

[B67] Rahman M. L. Feng Y. A. Fiehn O. Albert P. S. Tsai M. Y. Zhu Y. (2021). Plasma lipidomics profile in pregnancy and gestational diabetes risk: A prospective study in a multiracial/ethnic cohort. BMJ Open Diabetes Res. Care 9 (1), e001551. 10.1136/bmjdrc-2020-001551 PMC7939004 33674279

[B68] Rockman M. V. Kruglyak L. (2006). Genetics of global gene expression. Nat. Rev. Genet. 7 (11), 862–872. 10.1038/nrg1964 17047685

[B69] Rosenthal R. DiMatteo M. R. (2001). Meta-analysis: Recent developments in quantitative methods for literature reviews. Annu. Rev. Psychol. 52, 59–82. 10.1146/annurev.psych.52.1.59 11148299

[B70] Röst H. L. Sachsenberg T. Aiche S. Bielow C. Weisser H. Aicheler F. (2016). OpenMS: A flexible open-source software platform for mass spectrometry data analysis. Nat. Methods 13 (9), 741–748. 10.1038/nmeth.3959 27575624

[B71] Schmidt J. C. Dougherty B. V. Beger R. D. Jones D. P. Schmidt M. A. Mattes W. B. (2021). Metabolomics as a truly translational tool for precision medicine. Int. J. Toxicol. 40 (5), 413–426. 10.1177/10915818211039436 34514887 PMC8443142

[B72] Schrimpe-Rutledge A. C. Codreanu S. G. Sherrod S. D. McLean J. A. (2016). Untargeted metabolomics strategies-challenges and emerging directions. J. Am. Soc. Mass Spectrom. 27 (12), 1897–1905. 10.1007/s13361-016-1469-y 27624161 PMC5110944

[B73] Shaver A. O. Gouveia G. J. Kirby P. S. Andersen E. C. Edison A. S. (2021). Culture and Assay of Large-Scale Mixed-Stage <em&gt;*Caenorhabditis elegans*&lt;/em&gt; Populations. J. Vis. Exp. 2021 (171). 10.3791/61453 PMC12042146 34028439

[B74] Sherman E. Harbertson J. F. Greenwood D. R. Villas-Boas S. G. Fiehn O. Heymann H. (2018). Reference samples guide variable selection for correlation of wine sensory and volatile profiling data. Food Chem. 267, 344–354. 10.1016/j.foodchem.2017.10.073 29934177

[B75] Sindelar M. Stancliffe E. Schwaiger-Haber M. Anbukumar D. S. Adkins-Travis K. Goss C. W. (2021). Longitudinal metabolomics of human plasma reveals prognostic markers of COVID-19 disease severity. Cell. Rep. Med. 2 (8), 100369. 10.1016/j.xcrm.2021.100369 34308390 PMC8292035

[B76] Smirnoff N. (2018). Ascorbic acid metabolism and functions: A comparison of plants and mammals. Free Radic. Biol. Med. 122, 116–129. 10.1016/j.freeradbiomed.2018.03.033 29567393 PMC6191929

[B77] Smith C. A. Want E. J. O'Maille G. Abagyan R. Siuzdak G. (2006). Xcms: Processing mass spectrometry data for metabolite profiling using nonlinear peak alignment, matching, and identification. Anal. Chem. 78 (3), 779–787. 10.1021/ac051437y 16448051

[B78] Smith R. Ventura D. Prince J. T. (2015). LC-MS alignment in theory and practice: A comprehensive algorithmic review. Brief. Bioinform. 16 (1), 104–117. 10.1093/bib/bbt080 24273217

[B79] Soper-Hopper M. T. Vandegrift J. Baker E. S. Fernández F. M. (2020). Metabolite collision cross section prediction without energy-minimized structures. Analyst 145 (16), 5414–5418. 10.1039/d0an00198h 32583823 PMC7423765

[B80] Sousa S. A. A. Ferreira M. M. C. (2013). Optimized bucketing for NMR spectra: Three case studies. Chemom. Intelligent Laboratory Syst. 122 (122), 93–102. 10.1016/j.chemolab.2013.01.006

[B81] Spicer R. A. Salek R. Steinbeck C. (2017). Compliance with minimum information guidelines in public metabolomics repositories. Sci. Data 4, 170137. 10.1038/sdata.2017.137 28949328 PMC5613734

[B82] Stupp G. S. von Reuss S. H. Izrayelit Y. Ajredini R. Schroeder F. C. Edison A. S. (2013). Chemical detoxification of small molecules by *Caenorhabditis elegans* . ACS Chem. Biol. 8 (2), 309–313. 10.1021/cb300520u 23163740 PMC3747836

[B83] Sumner L. W. Amberg A. Barrett D. Beale M. H. Beger R. Daykin C. A. (2007). Proposed minimum reporting standards for chemical analysis chemical analysis working group (CAWG) metabolomics standards initiative (MSI). Metabolomics 3 (3), 211–221. 10.1007/s11306-007-0082-2 24039616 PMC3772505

[B84] Tautenhahn R. Böttcher C. Neumann S. (2008). Highly sensitive feature detection for high resolution LC/MS. BMC Bioinforma. 9 (1), 504. 10.1186/1471-2105-9-504 PMC2639432 19040729

[B85] Tomasi G. Andersson C. (2004). Correlation optimized warping and dynamic time warping as preprocessing methods for chromatographic data. J. Chemom. 18 (5), 231–241. 10.1002/cem.859

[B86] Ulrich E. L. Akutsu H. Doreleijers J. F. Harano Y. Ioannidis Y. E. Lin J. (2008). Nucleic Acids Res. 36, D402–D408. 10.1093/nar/gkm957 17984079 PMC2238925

[B87] van der Sijde M. R. Ng A. Fu J. (2014). Systems genetics: From GWAS to disease pathways. Biochim. Biophys. Acta 1842 (10), 1903–1909. 10.1016/j.bbadis.2014.04.025 24798234

[B88] Viechtbauer W. (2010). Conducting meta-analyses in R with the metafor package. J. Stat. Softw. 36 (3), 48. 10.18637/jss.v036.i03

[B89] Walejko J. M. Chelliah A. Keller-Wood M. Gregg A. Edison A. S. (2018). Global metabolomics of the placenta reveals distinct metabolic profiles between maternal and fetal placental tissues following delivery in non-labored women. Metabolites 8 (1), E10. 10.3390/metabo8010010 PMC5876000 29360753

[B90] Wasito H. Hermann G. Fitz V. Troyer C. Hann S. Koellensperger G. (2021). Yeast-based reference materials for quantitative metabolomics. Anal. Bioanal. Chem. 414, 4359–4368. 10.1007/s00216-021-03694-w 34642781 PMC9142427

[B91] Wehrens R. Hageman J. A. van Eeuwijk F. Kooke R. Flood P. J. Wijnker E. (2016). Improved batch correction in untargeted MS-based metabolomics. Metabolomics. 12, 88. 10.1007/s11306-016-1015-8 27073351 PMC4796354

[B92] Wishart D. S. Guo A. Oler E. Wang F. Anjum A. Peters H. (2022). Hmdb 5.0: The human metabolome database for 2022. Nucleic Acids Res. 50 (D1), D622–D631. 10.1093/nar/gkab1062 34986597 PMC8728138

[B93] Wong J. W. Durante C. Cartwright H. M. (2005). Application of fast Fourier transform cross-correlation for the alignment of large chromatographic and spectral datasets. Anal. Chem. 77 (17), 5655–5661. 10.1021/ac050619p 16131078

[B94] Wulff Jem M. W. Mitchell M. W. (2018). A comparison of various normalization methods for LC/MS metabolomics data. Adv. Biosci. Biotechnol. 9, 339–351. 10.4236/abb.2018.98022

[B95] Xi Y. Rocke D. M. (2008). Baseline correction for NMR spectroscopic metabolomics data analysis. BMC Bioinforma. 9, 324. 10.1186/1471-2105-9-324 PMC2516527 18664284

[B96] Yang N. Sun R. Liao X. Aa J. Wang G. (2017). UDP-glucuronosyltransferases (UGTs) and their related metabolic cross-talk with internal homeostasis: A systematic review of UGT isoforms for precision medicine. Pharmacol. Res. 121, 169–183. 10.1016/j.phrs.2017.05.001 28479371

[B97] Yilmaz L. S. Walhout A. J. (2016). A *Caenorhabditis elegans* genome-scale metabolic network model. Cell. Syst. 2 (5), 297–311. 10.1016/j.cels.2016.04.012 27211857 PMC5387690

[B98] Yoshimura J. Ichikawa K. Shoura M. J. Artiles K. L. Gabdank I. Wahba L. (2019). Recompleting the *Caenorhabditis elegans* genome. Genome Res. 29 (6), 1009–1022. 10.1101/gr.244830.118 31123080 PMC6581061

[B99] Zhang F. Robinette S. L. Bruschweiler-Li L. Bruschweiler R. (2009). Web server suite for complex mixture analysis by covariance NMR. Magn. Reson. Chem. 47 (1), S118–S122. 10.1002/mrc.2486 19634130 PMC2865847

[B100] Zhang G. Mostad J. D. Andersen E. C. (2021). Natural variation in fecundity is correlated with species-wide levels of divergence in *Caenorhabditis elegans* . G3 (Bethesda) 11 (8), jkab168. 10.1093/g3journal/jkab168 33983439 PMC8496234

