# Supplementary material for: An anchored experimental design and meta-analysis approach to address batch effects in large-scale metabolomics
Source: Front Mol Biosci. 2022 Nov 9;9:930204. doi: 10.3389/fmolb.2022.930204 (PMC9682135; doi:10.3389/fmolb.2022.930204)
Supplement: Supplementary file 1 [file DataSheet1.pdf]

SUPPLEMENTARY INFORMATION

Growth of C. elegans using the LSCP method yields on average 2.4 million mixed-stage worms per sample

Growth of *C. elegans* using the LSCP method generates large mixed-stage populations of *C. elegans* with minor handling and manipulation of the animals is ideal for metabolomics experiments (43). Population dynamics depend on the strain's behavior (*i.e*., burrowing strains tend to have lower worm recovery) and growth success (*i.e.*, contamination). The LSCP method yields population sizes from approximately 94,500 to 9,290,000. The mean population size within the reference strain, PD1074, and across strains is approximately 2.4 million worms (**Figure S1**). PD1074 LSCPs take between 10 – 14 days to grow to a full mixed-stage population. The mean growth time for PD1074 is ten days. The slowest growing strain grows for a maximum of 20 days, and the fastest growing strain for a minimum of 10 days (**Figure S1**). Strain-dependent variation in growth rate is to be expected because of the wide range of strains and traits included in this study. Notably, growth time is not indicative of the final population size (**Figure S1**). Additional information on *C. elegans* growth and population dynamics has been described previously (43).

Reversed Phase (RP) chromatography method

Non-polar extracts were separated using a Vanquish liquid chromatograph (ThermoFisher Scientific), fitted with a ThermoFisher Scientific Accucore™ C30 UPLC RP column (2.1 x 150 mm, 2.6 µm particle size). The compounds were eluted with the following gradient: 60:40 acetonitrile:water (ACN:H_2O) with 10 mM ammonium formate and 0.1% formic acid (mobile phase A) and 90:10 isopropanol:acetonitrile with 10 mM ammonium formate and 0.1% formic acid (mobile phase B) using the following gradient program: 0.0 min 20% B; 1.0 min 60% B; 5.0 min 70% B; 5.5 min 85% B; 8.0 min 90% B; 8.2-10.5 min 100% B; 10.7-12.0 min 20% B. A curve 5 value was set for 0.0 minutes, and a curve 6 for the remainder of the gradient. The flow rate was set at 0.400 mL min^{-1}. The column temperature was set to 50°C, and the injection volume was 2 µL. The following internal standards were spiked in for RP analysis: 15:0-18:1(d7) PC, 15:0-18:1(d7) PE, 15:0-, 18:1(d7) PS, 15:0-18:1(d7) PG, 15:0-18:1(d7) PI, 18:1(d7) LPC, 18:1(d7) LPE, 18:1(d7) Chol Ester, 15:0-18:1(d7) DG, 15:0-18:1(d7)-15:0 TG, 18:1(d9) SM, and Cholesterol (d7).

Hydrophilic Interaction Liquid Chromatography (HILIC) method

Polar extracts were separated using a Vanquish liquid chromatograph (ThermoFisher Scientific), fitted with a Waters Acquity UPLC BEH Amide column (2.1 x 150 mm, 1.7 µm particle size). The compounds were eluted with the following gradient: 80:20 water:acetonitrile (H_2O:ACN) with 10 mM ammonium formate and 0.1% formic acid (mobile phase A) and 100% ACN with 0.1% formic acid (mobile phase B) using the following gradient program: 0.0-0.5 min 95% B; 8.0-9.4 min 40% B; 9.5-11.0 min 95% B. A curve 5 value was set for 0.0 minutes, a curve 6 at 0.5 min, curve 7 at 8.0 min, and a curve 6 for the remainder of the gradient. The flow rate was set at 0.400 mL min^{-1}. The column temperature was set to 40°C, and the injection volume was 2 µL.

Mass spectrometer settings and methods

An Orbitrap ID-X Tribrid mass spectrometer (ThermoFisher Scientific) equipped with a HESI ion source was used for all mass spectrometry data collection. For HILIC analysis the mass spectrometer was run in full MS mode at a resolution of 240,000 (FWHM at *m/z* 200) for the duration of the chromatographic gradient. A normalized automatic gain control (AGC) target of 100% was set with a maximum injection time of 100 ms. A tune file with the following source conditions was used for positive and negative mode: spray voltage (+) 3500, spray volage (-) 2500, vaporizer temperature: 275 ºC, sheath gas: 40, aux gas: 8, sweep gas: 1, and S-Lens RF level: 60%. Scan range covered *m/z* 70-1050. Calibration was conducted using ThermoFisher Pierce™ Negative Ion Calibration Solution and Pierce™ LTQ Velos ESI Positive Ion Calibration Solution prior to collecting negative and positive mode data, respectively.

Identical parameters were used for reversed-phase analysis with the following exceptions: spray voltage (+) 3500, spray volage (-) 2800, maximum injection time of 200ms, vaporizer temperature: 425 ºC, sheath gas: 60, aux gas: 18, sweep gas: 4, and a scan range of *m/z* 150-2000.

Variation between PD1074 and N2

The reference strain PD1074, obtained from CeNDR, is a traceable variant of the laboratory-adapted N2 Bristol strain (101). Here, we include N2, obtained from CeNDR, as one of our natural strains as an additional way to validate our methods and processes. Phenotypically, PD1074 is not significantly different in the amount of time needed to cultivate a population or the

resulting population size from N2 (**Figure S1**). Metabolically, PD1074 is very similar to N2 showing only 16 total feature differences in the RP LC-MS (+) data, six feature differences in the RP LC-MS (-), and seven differences in the HILIC LC-MS (+) data (**Table 1).** PD1074 and N2 show three and seven significant feature differences in the NMR non-polar polar data, respectively (**Table 1**). Both the phenotypic and metabolic data demonstrate that these strains are very similar, as expected based on their genetic relationship. In contrast, in the RP LC-MS modes, N2 has up to a 50-fold difference from the other strains (**Figure S6**).

SUPPLEMENTARY FIGURES

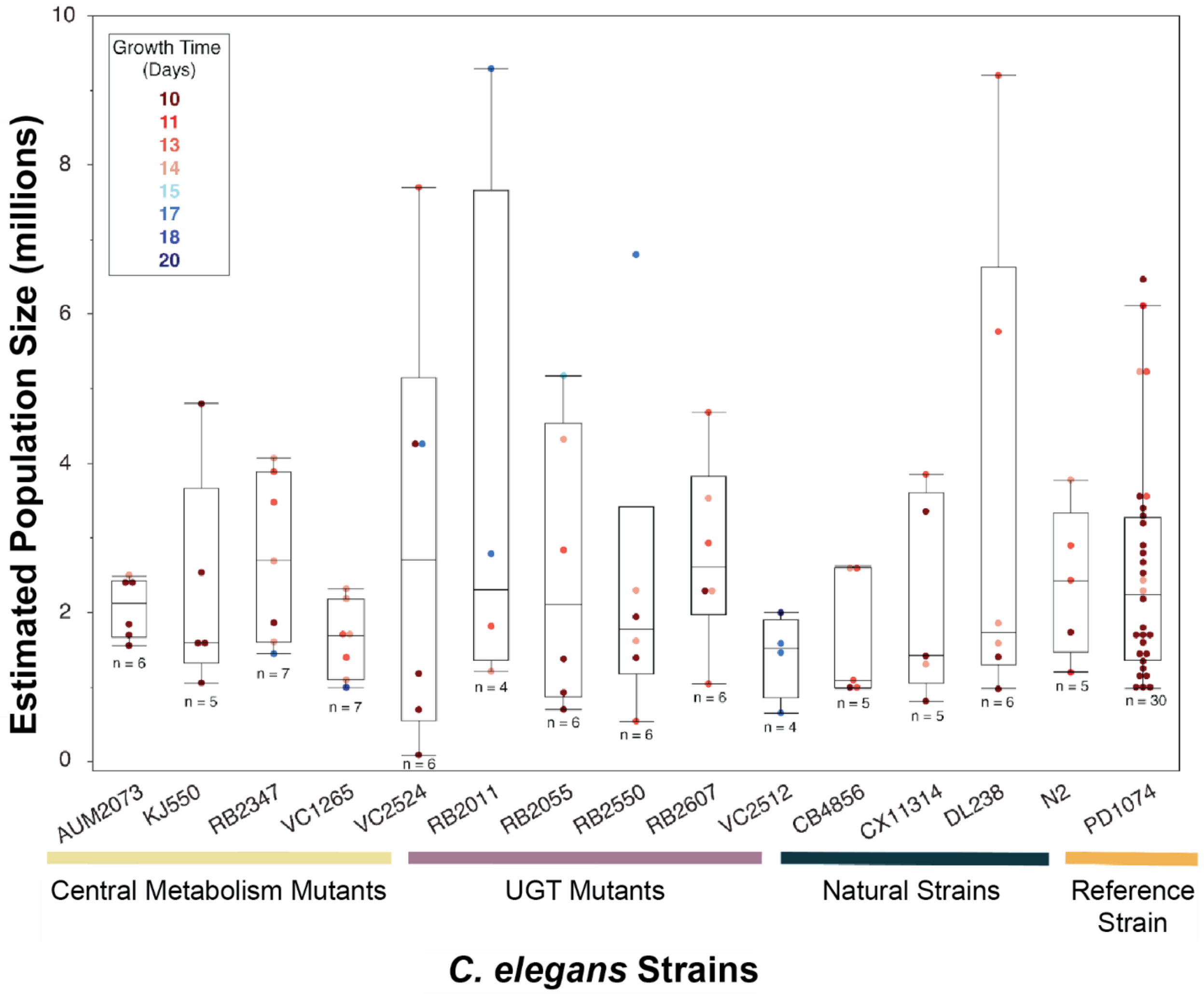

Supplementary Figure 1. **LSCP method generates, on average, a population of 2.4 million mixed-stage *C. elegans* populations.** The LSCP yields population sizes in the smallest population growths at around 94,500 worms and the biggest population growths at around 9,290,000 worms. The mean population size across all strains was 2.4 million worms. Bars underneath *C. elegans* strain names indicate each strains study. Comparisons of population size for all pairs using Tukey's HSD test were performed. No significant differences are observed between estimated population sizes across *C. elegans* strains. Colored data points indicate the growth time (days) to generate a given LSCP sample.

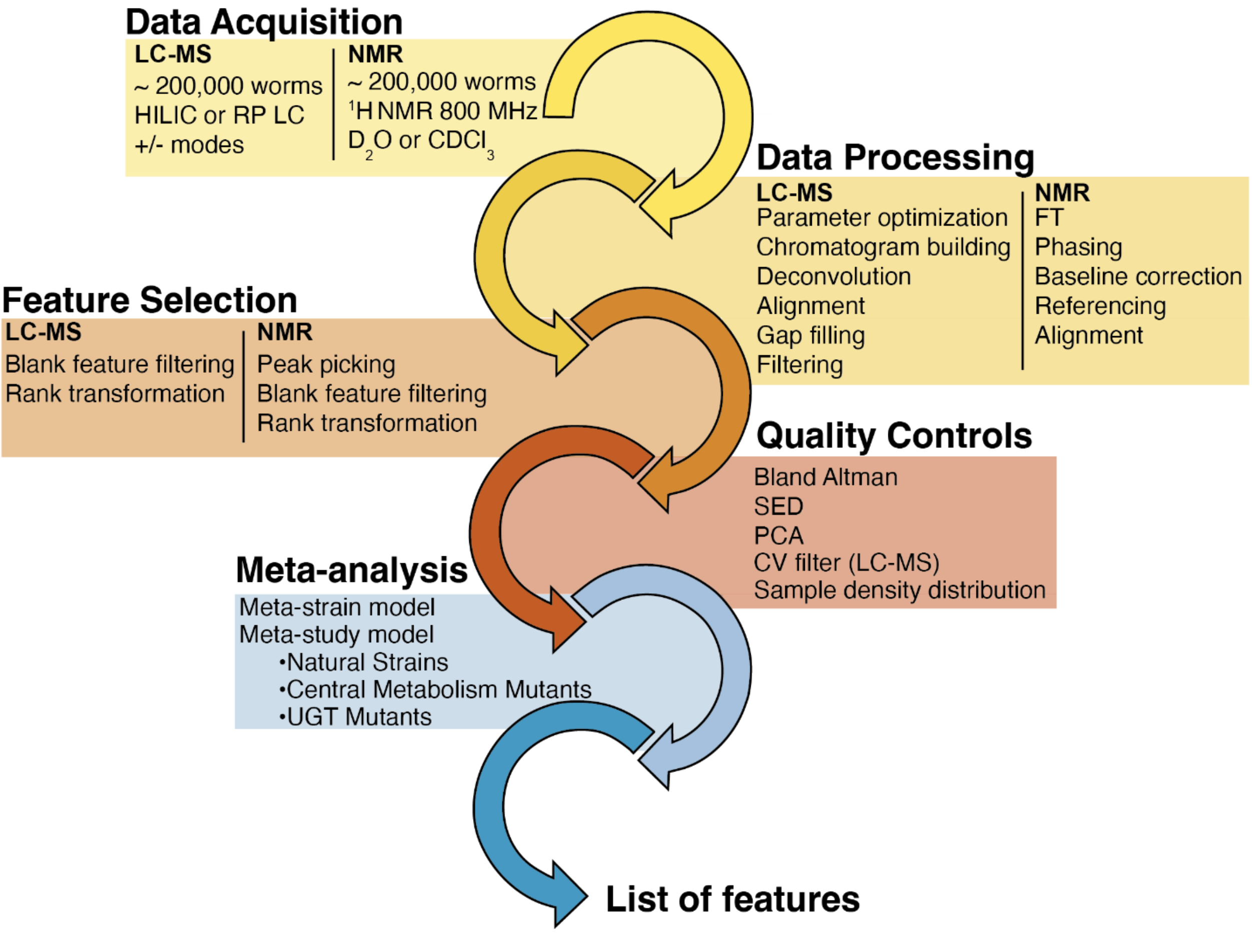

Supplementary Figure 2. Unified workflow for spectral feature selection across platforms. Steps and software listed to obtain spectral features. LC-MS-specific steps are listed on the left side of each process. NMR-specific steps are denoted on the right side of each process. When neither NMR nor LC-MS is indicated, the same steps are performed on data from both analytical platforms.

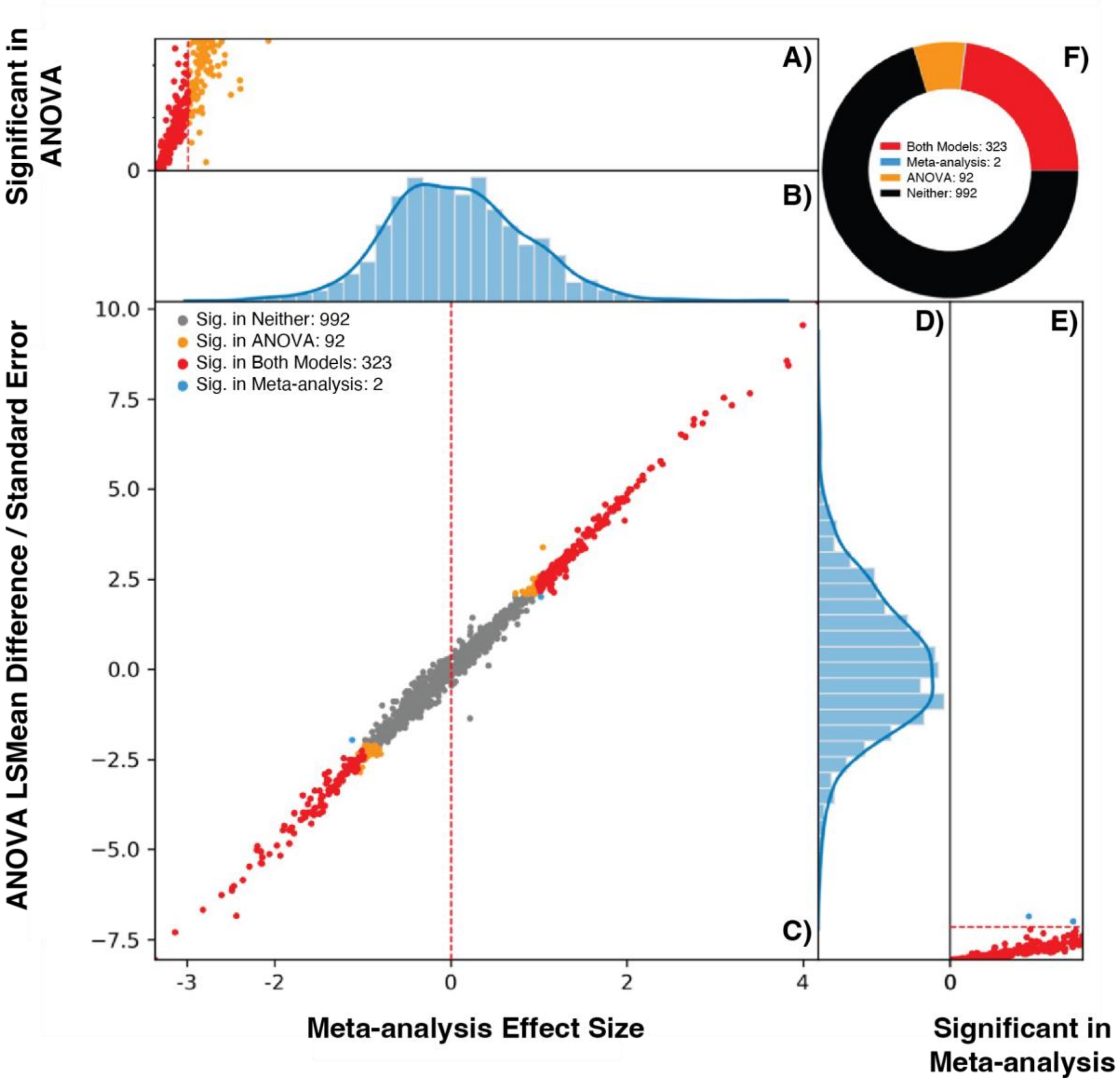

Supplementary Figure 3: Comparing the inferences from a linear model and meta-analysis using the test genotype, VC1265. There are six panels in the plot. **(A)** Displays the p-values where the y-axis is from 0-1 and represents the significant results (p-value < 0.05) in the linear model. **(B)** Displays the effect size distribution for effect sizes in the x-axis for the scatter plot. **(C)** Displays a scatter plot, where the x-axis is the effect sizes calculated by the meta-analysis model and the y-axis is the *lsmean* difference calculation. Each point is one spectral feature. **(D)** Displays the effect size distribution for effect sizes in the y-axis for the scatter plot. Point colors represent significance of the test of the null hypothesis where the mean peak height for VC1265 is not different from the mean peak height of PD1074 with a nominal threshold p-value < 0.05. Red points are significant in both models. Orange points are significant in the linear model. Blue points are significant results in the meta-analysis model. Grey points indicates that spectral feature is not significant in either model. Red lines in **(A)** and **(E)** are significance thresholds for the nominal p-value < 0.05 on the other test. **(F)** Summary of the results.

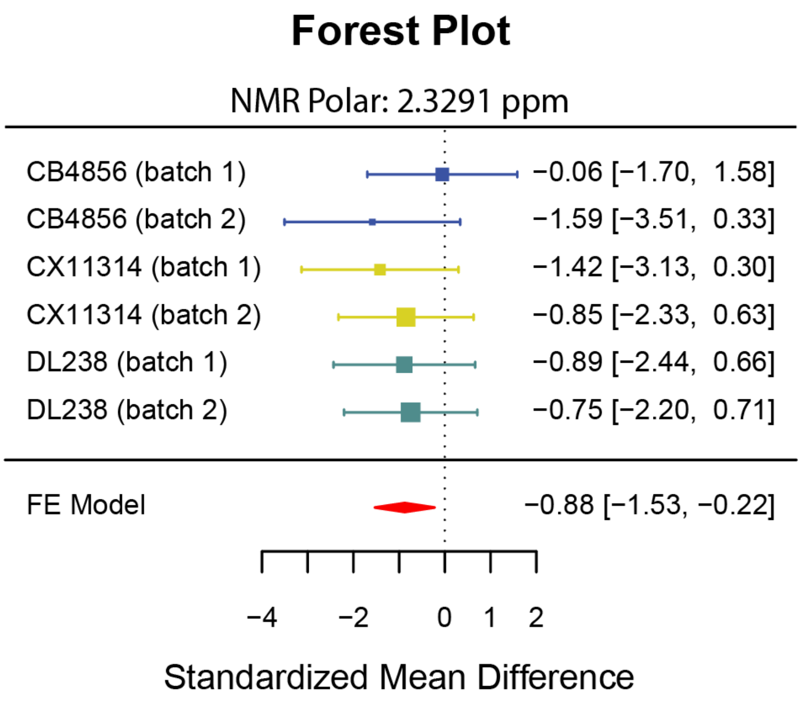

Supplementary Figure 4. Forest plot comparing the meta-strain and meta-study models in the NS for the NMR polar feature 2.3291 ppm. The meta-strain model for each NS in each batch is displayed, where the area of each square is proportional to the study's weight in the meta-analysis with confidence intervals (CI) represented by whiskers. The dashed vertical line represents the overall measure of effect. The right-hand column is the measure of effect (odds ratio) for each study. The meta-study model (FE model) is represented by the red diamond, where the lateral points indicate CI for the estimate.

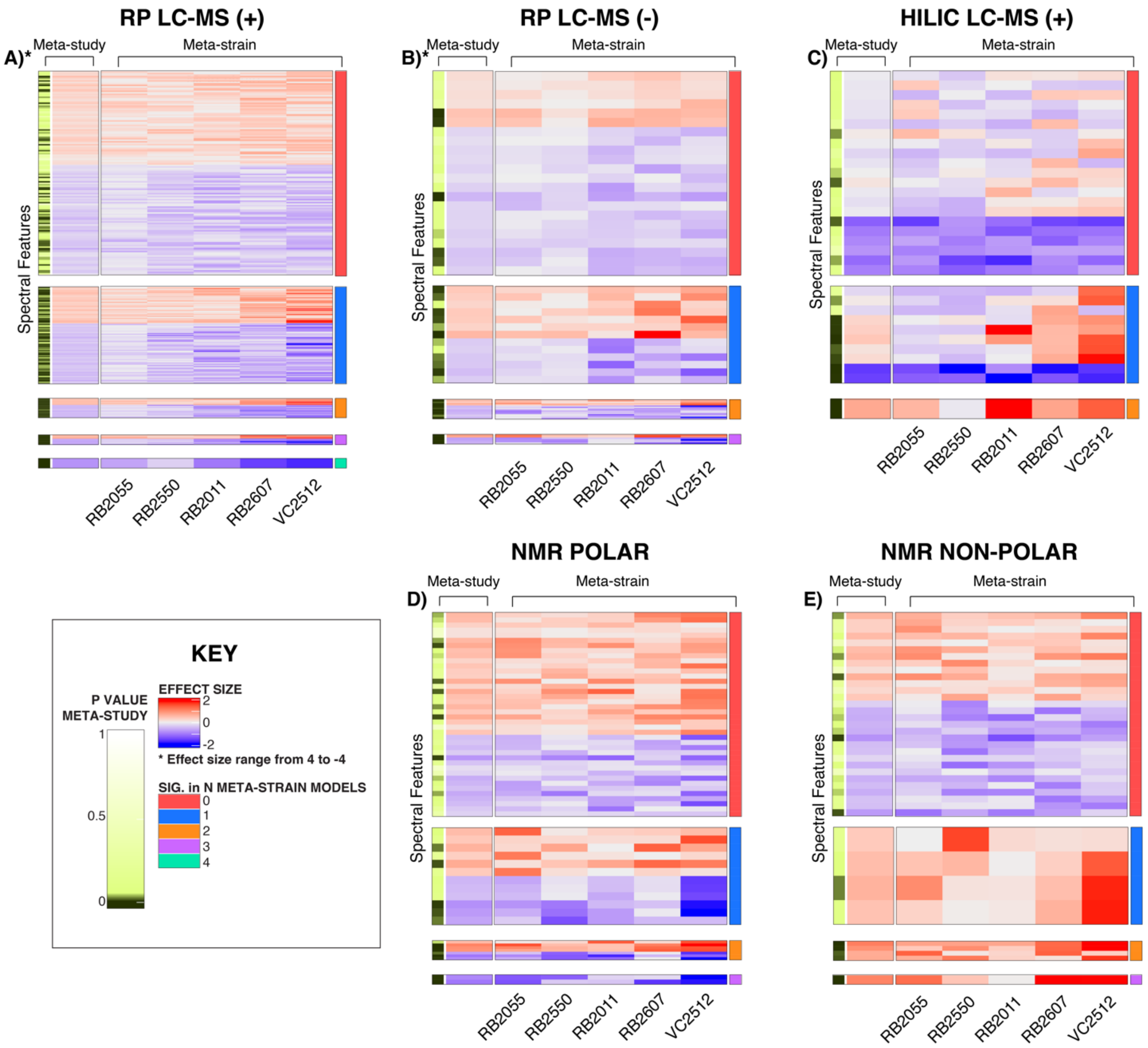

Supplementary Figure 5. Heatmaps of significant spectral features in the UGT mutant study. A) RP LC-MS positive mode **(B)** RP LC-MS negative mode **(C)** HILIC LC-MS positive mode **(D)** NMR polar and **(E)** NMR non-polar. The first two columns pertain to the meta-study results. The left-most column indicates the significant features found in the meta-study model, followed by the estimated effect size in the meta-study model across CM mutants. The following five columns are the effect sizes for individual strains, and the contents of the cell are the estimates of the effect of that strain (column), for that feature (row) compared to PD1074 from the meta-strain model. Features where the effect sizes are consistent for all strains are included. The effect sizes range from (2 to -2) for HILIC LC-MS positive mode, NMR polar, and NMR non-polar. The effect sizes range from (4 to -4) for RP LC-MS positive mode and RP LC-MS negative mode, denoted by asterisks. Positive effect sizes (*i.e.*, the strain had a higher peak at that given metabolic feature than PD1074) are displayed in red. Negative effect sizes (*i.e.,* PD1074 had a higher peak at that given metabolic feature than the test strain) are displayed in blue. The rightmost column indicates the number of strains in which a given spectral feature is statistically significant.

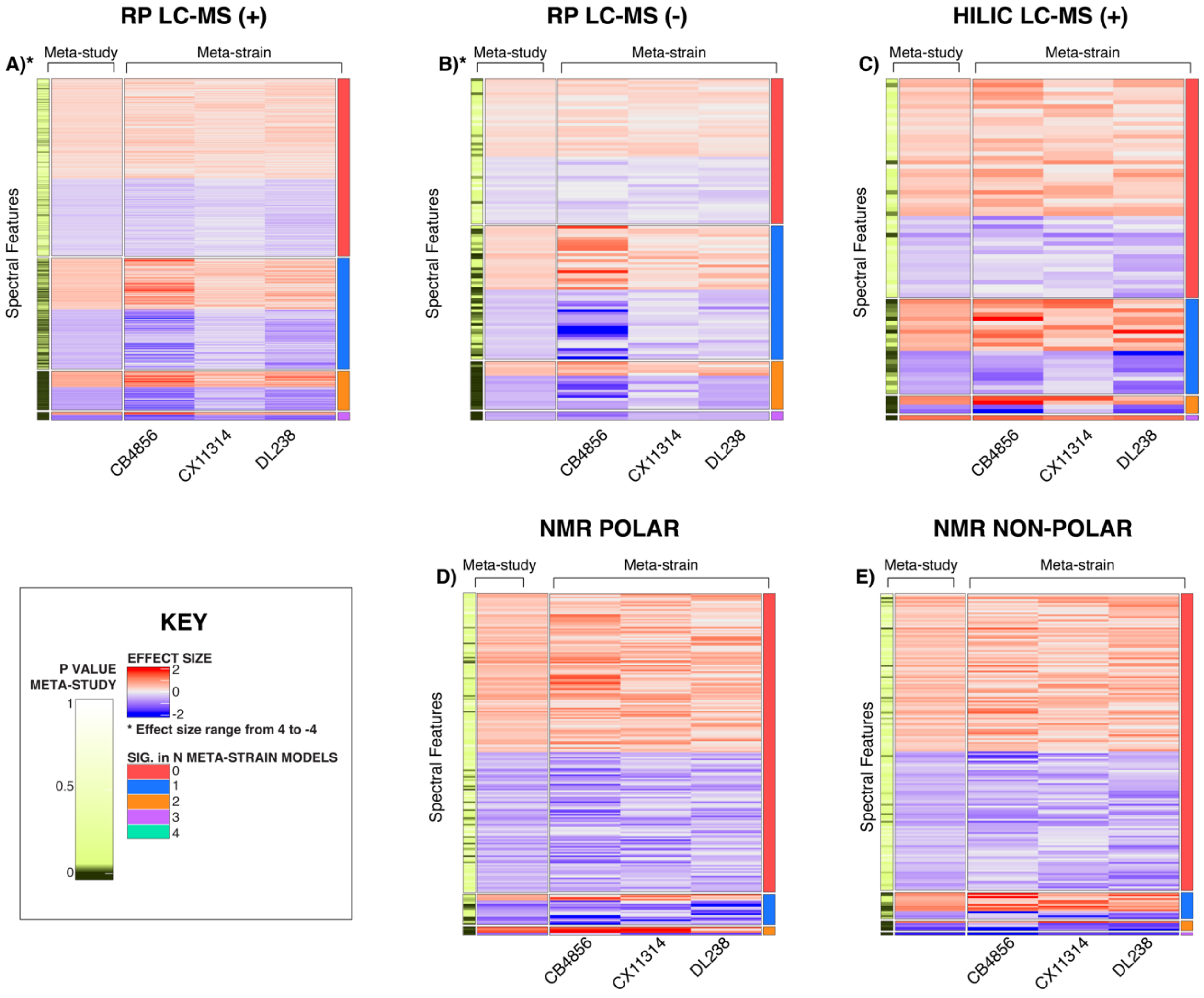

Supplementary Figure 6. Heatmaps of significant spectral features identified in the NS study. A) RP LC-MS positive mode **(B)** RP LC-MS negative mode **(C)** HILIC LC-MS positive mode **(D)** NMR polar and **(E)** NMR non-polar. The first two columns pertain to the meta-study results. The left-most column indicates the significant features found in the meta-study model, followed by the estimated effect size in the meta-study model across CM mutants. The following five columns are the effect sizes for individual strains, and the contents of the cell are the estimates of the effect of that strain (column), for that feature (row) compared to PD1074 from the meta-strain model. Features where the effect sizes are consistent for all strains are included. The effect sizes range from (2 to -2) for HILIC LC-MS positive mode, NMR polar, and NMR non-polar. The effect sizes range from (4 to -4) for RP LC-MS positive mode and RP LC-MS negative mode, denoted by asterisks. Positive effect sizes (*i.e.*, the strain had a higher peak at that given metabolic feature than PD1074) are displayed in red. Negative effect sizes (*i.e.,* PD1074 had a higher peak at that given metabolic feature than the test strain) are displayed in blue. The rightmost column indicates the number of strains in which a given spectral feature is statistically significant.

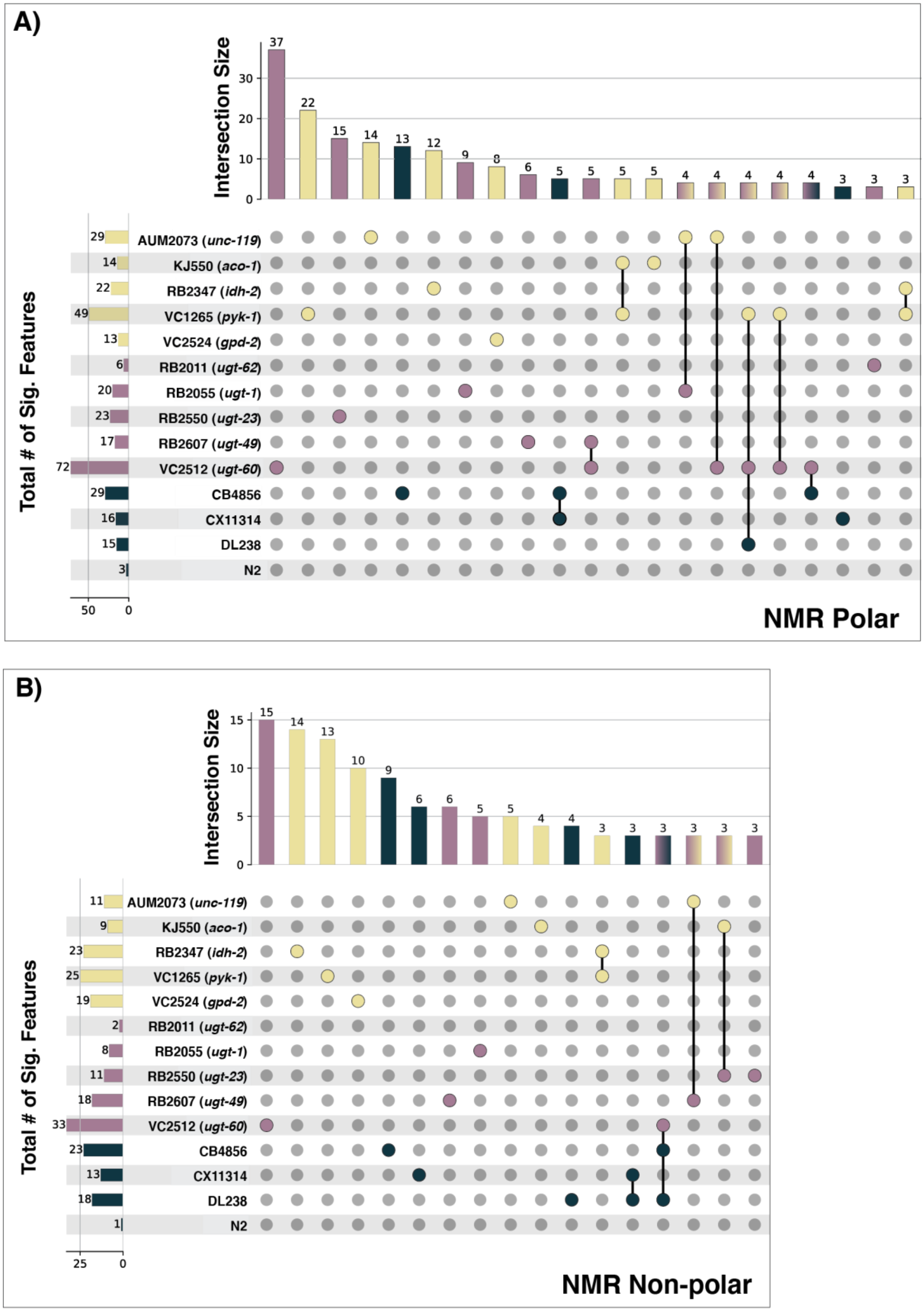
A)
Intersection Size
37
22
15
14
13
12
9
8
6
5
5
5
5
4
4
4
4
4
3
3
3
Total # of Sig. Features
AUM2073 (unc-119)
KJ550 (aco-1)
RB2347 (idh-2)
VC1265 (pyk-1)
VC2524 (gpd-2)
RB2011 (ugt-62)
RB2055 (ugt-1)
RB2550 (ugt-23)
RB2607 (ugt-49)
VC2512 (ugt-60)
CB4856
CX11314
DL238
N2
29
14
22
49
13
6
20
23
17
72
29
16
15
3
NMR Polar
B)
Intersection Size
15
14
13
10
9
6
6
5
5
4
4
3
3
3
3
3
3
Total # of Sig. Features
AUM2073 (unc-119)
KJ550 (aco-1)
RB2347 (idh-2)
VC1265 (pyk-1)
VC2524 (gpd-2)
RB2011 (ugt-62)
RB2055 (ugt-1)
RB2550 (ugt-23)
RB2607 (ugt-49)
VC2512 (ugt-60)
CB4856
CX11314
DL238
N2
11
9
23
25
19
2
8
11
18
33
23
13
18
1
NMR Non-polar

Supplementary Figure 7. Significant features in NMR data for the meta-strain model. For features significant in at least one strain. (**A)** NMR polar (**B)** NMR non-polar. Fourteen strains in the CM mutants (yellow), UGT mutants (purple), and NS (green) are displayed. Horizontal bar plots sum the total number of significant features for a strain. Vertical bar plots sum significant feature interactions within and across strains. Significant feature connections below three are not displayed.

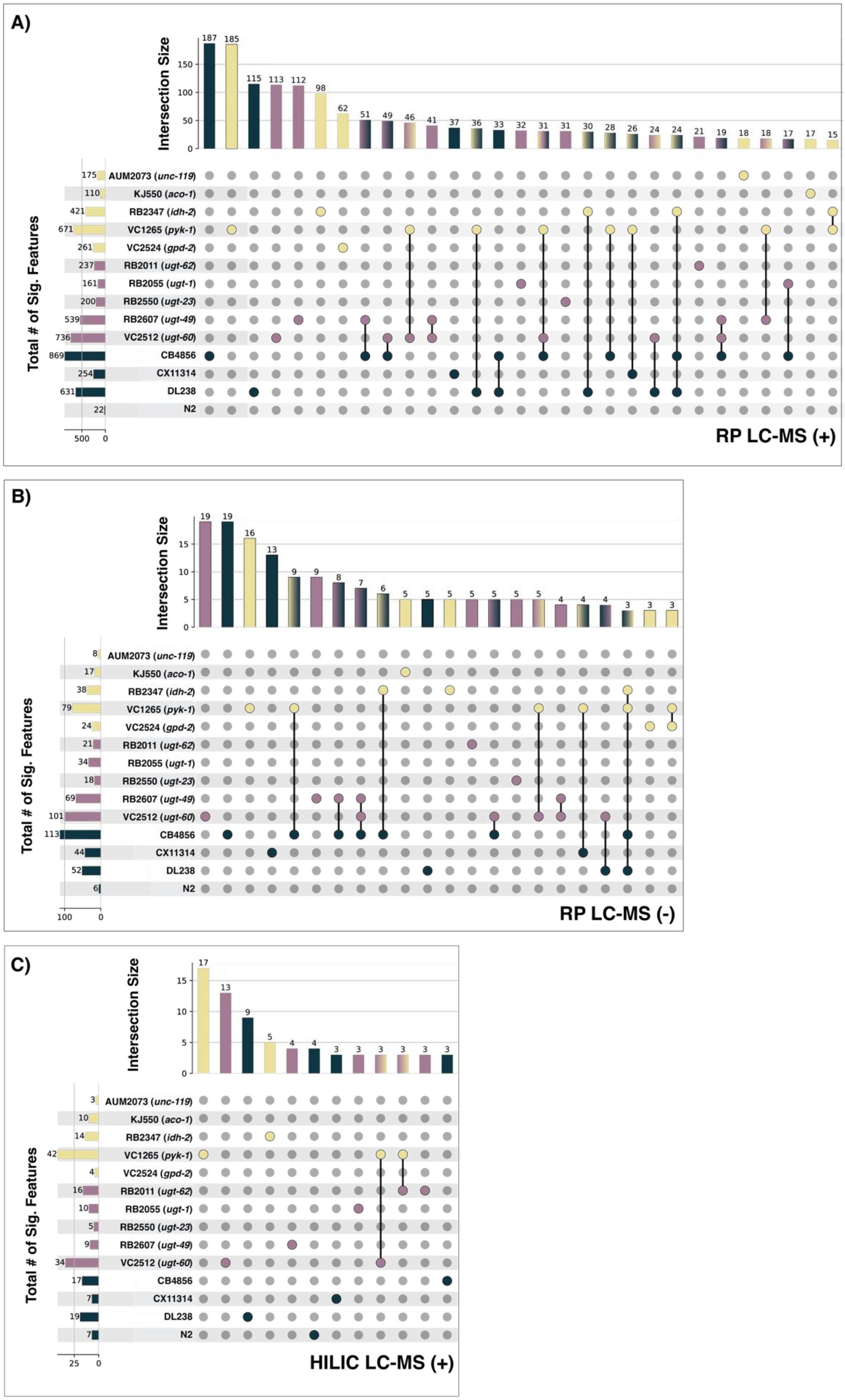
A)
Intersection Size
187 185 115 113 112 98 62 51 49 46 41 37 36 33 32 31 31 30 28 26 24 24 21 19 18 18 17 17 15
Total # of Sig. Features
175 AUM2073 (unc-119)
110 KJ550 (aco-1)
421 RB2347 (idh-2)
671 VC1265 (pyk-1)
261 VC2524 (gpd-2)
237 RB2011 (ugt-62)
161 RB2055 (ugt-1)
200 RB2550 (ugt-23)
539 RB2607 (ugt-49)
736 VC2512 (ugt-60)
869 CB4856
254 CX11314
631 DL238
22 N2
RP LC-MS (+)
B)
Intersection Size
19 19 16 13 9 9 8 7 6 5 5 5 5 5 5 5 4 4 4 3 3 3
Total # of Sig. Features
8 AUM2073 (unc-119)
17 KJ550 (aco-1)
38 RB2347 (idh-2)
79 VC1265 (pyk-1)
24 VC2524 (gpd-2)
21 RB2011 (ugt-62)
34 RB2055 (ugt-1)
18 RB2550 (ugt-23)
69 RB2607 (ugt-49)
101 VC2512 (ugt-60)
113 CB4856
44 CX11314
52 DL238
6 N2
RP LC-MS (-)
C)
Intersection Size
17 13 9 5 4 4 3 3 3 3 3 3
Total # of Sig. Features
3 AUM2073 (unc-119)
10 KJ550 (aco-1)
14 RB2347 (idh-2)
42 VC1265 (pyk-1)
4 VC2524 (gpd-2)
16 RB2011 (ugt-62)
10 RB2055 (ugt-1)
5 RB2550 (ugt-23)
9 RB2607 (ugt-49)
34 VC2512 (ugt-60)
17 CB4856
7 CX11314
19 DL238
7 N2
HILIC LC-MS (+)

Supplementary Figure 8. Significant features found via the meta-strain model across the three study groups in LC-MS. For features significant in at least one strain. **(A)** RP LC-MS positive mode (**B)** RP LC-MS negative mode (**C)** HILIC LC-MS positive mode. Fourteen strains in the CM mutants (yellow), UGT mutants (purple), and NS (green) are displayed. Horizontal bar plots sum the total number of significant features for a strain. Vertical bar plots sum significant feature interactions within and across strains. Significant feature connections below three are not displayed for all modes, except for the RP LC-MS positive mode, where connections below 15 are not displayed.

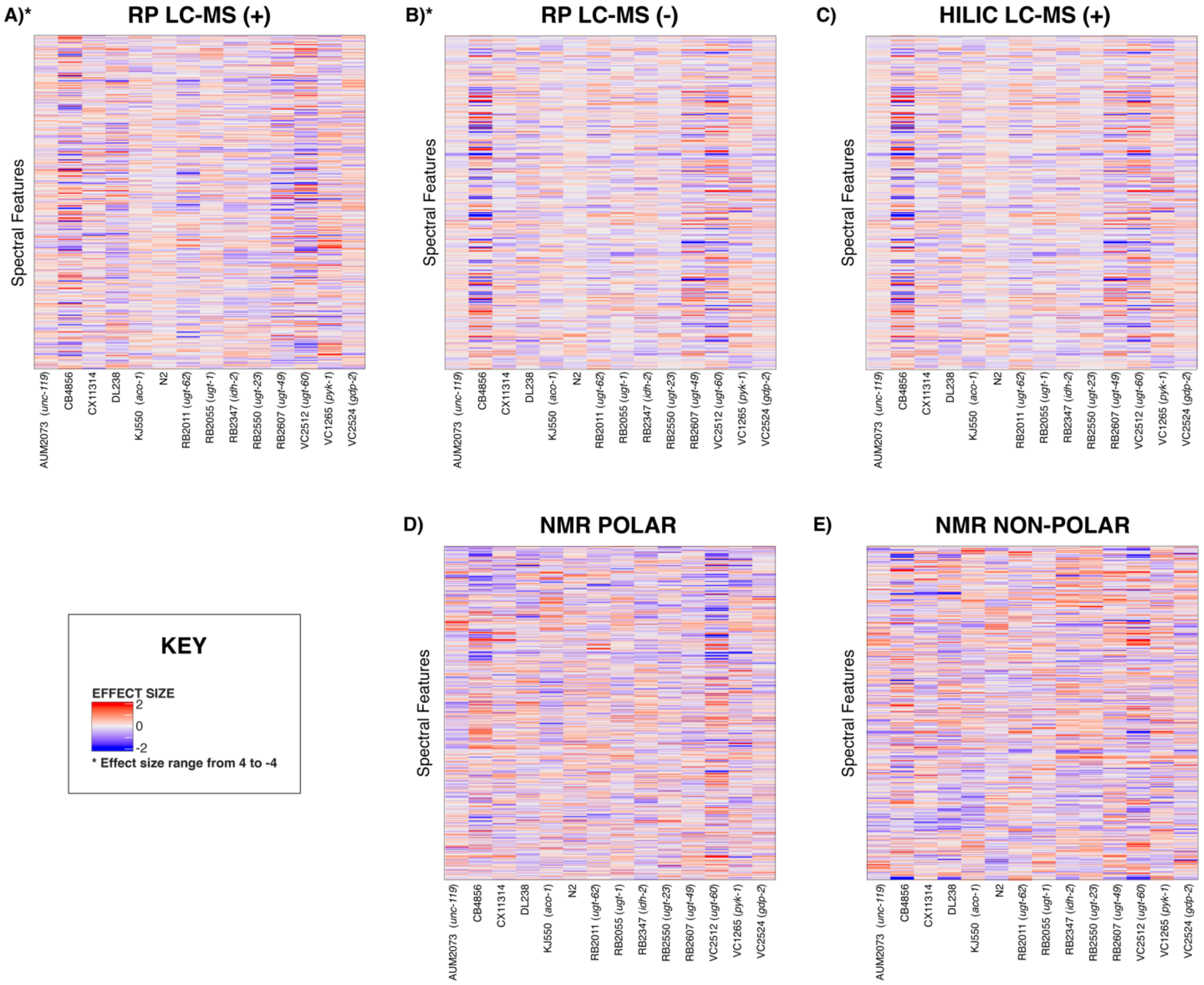

Supplementary Figure 9. Heatmaps of stable spectral features identified via the meta-strain model across all test strains. A) RP LC-MS positive mode **(B)** RP LC-MS negative mode **(C)** HILIC LC-MS positive mode **(D)** NMR polar and **(E)** NMR non-polar. Each column represents an individual strain, and the contents of the cell are the estimates of the effect of that strain, for that feature compared to PD1074 using the meta-analytic approach (the 'meta-strain' model). For each panel, a row represents a spectral feature with an effect size that is significantly different in at least one strain, and consistently higher or lower relative to PD1074. The effect sizes range from (2 to -2). Modes denoted with an asterisk have effect sizes that range from (4 to -4). Positive

effect sizes (*i.e*., the strain had a higher peak at that given metabolic feature than PD1074) are displayed in red. Negative effect sizes (*i.e.,* PD1074 had a higher peak at that given metabolic feature than the test strain) are displayed in blue.

SUPPLEMENTARY TABLES

Strain	Genotype	Source	About	Study Group
PD1074	wild type	CeNDR	wild type	Anchor Strain
AUM2073	*unc-119; vizSi34 II; unc-119(ed3) III.*	CGC	GSK-3 promotes S-phase entry and progress in germline cells to maintain tissue output	Central Metabolism Mutants
KJ550	*aco-1 (jh131) X.*		Exhibits aconitate hydratase activity. Is involved in tricarboxylic acid metabolic process. Localizes to cytosol. Is expressed in seam cell. Is an ortholog of human ACO1 (aconitase 1).	
RB2347	*idh-2(ok3183) X.*		Is predicted to have isocitrate dehydrogenase (NADP+) activity and metal ion binding activity. Human ortholog(s) of this gene are implicated in D-2-hydroxyglutaric aciduria 2. Is an ortholog of human IDH2 (isocitrate dehydrogenase (NADP(+)) 2).	
VC1265	*pyk-1; F25H5.3(ok1754) I.*		*pyk-1 encodes for one of two pyruvate kinases. essential for embryonic development, Is predicted to have kinase activity; metal ion binding activity; and pyruvate kinase activity. Human ortholog(s) of this gene are implicated in pyruvate kinase deficiency of red cells. Is an ortholog of human PKLR (pyruvate kinase L/R) and PKM (pyruvate kinase M1/2).*	
VC2524	*gpd-2 (ok3243) X.*		Is predicted to have NAD binding activity; NADP binding activity; and glyceraldehyde-3-phosphate dehydrogenase (NAD+) (phosphorylating) activity. Is expressed in several structures, including AB; Psub1; and head. Is an ortholog of human GAPDH (glyceraldehyde-3-phosphate dehydrogenase).	
RB2011	*ugt-62 (ok2663)*	CGC	homozygous. gene knockout ugt-62, Is predicted to have glucuronosyltransferase activity. Human ortholog(s) of this gene are implicated in Crigler-Najjar syndrome and Gilbert syndrome. Is an ortholog of several human genes including UGT1A6 (UDP glucuronosyltransferase family 1 member A6); UGT1A8 (UDP glucuronosyltransferase family 1 member A8); and UGT1A9 (UDP glucuronosyltransferase family 1 member A9).	UGT Mutants
RB2055	*ugt-1 (ok2718) V.*		homozygous. gene knockout ugt-1, Is predicted to have UDP-glycosyltransferase activity. Human ortholog(s) of this gene are implicated in Crigler-Najjar syndrome and Gilbert syndrome. Is an ortholog of several human genes including UGT1A4 (UDP glucuronosyltransferase family 1 member A4); UGT1A8 (UDP glucuronosyltransferase family 1 member A8); and UGT1A9 (UDP glucuronosyltransferase family 1 member A9).	
RB2550	*ugt-23 (ok3541) X.*		homozygous. gene knockout ugt-23, Is predicted to have glucuronosyltransferase activity. Is involved in gastrulation. Human ortholog(s) of this gene are implicated in Crigler-Najjar syndrome and Gilbert syndrome. Is an ortholog of human UGT3A1 (UDP glycosyltransferase family 3 member A1) and UGT3A2 (UDP glycosyltransferase family 3 member A2).	
RB2607	*ugt-49(ok3633) V.*		homozygous. gene knockout ugt-49, Is predicted to have glucuronosyltransferase activity. Human ortholog(s) of this gene are implicated in Crigler-Najjar syndrome and Gilbert syndrome. Is an ortholog of several human genes including UGT2A3 (UDP glucuronosyltransferase family 2 member A3); UGT2B10 (UDP glucuronosyltransferase family 2 member B10); and UGT2B11 (UDP glucuronosyltransferase family 2 member B11).	
VC2512	*ugt-60(ok3248) III/hT2 [bli-4(e937) let-?(q782) qIs48] (I;III).*		homozygous. gene knockout ugt-60, Is predicted to have glucuronosyltransferase activity. Human ortholog(s) of this gene are implicated in Crigler-Najjar syndrome and Gilbert syndrome. Is an ortholog of human UGT2B7 (UDP glucuronosyltransferase family 2 member B7).	
N2	wild type	CeNDR	wild type	Natural Strains
DL238				
CX11314				
CB4856				

Supplementary Table 1. *C. elegans* genotypes used in study.

SECIM Tools Workflow:	Input Parameters	Set Value
Blank Feature Filtering (BFF)	BFF Threshold	5000
	Criterion Value	100
Standard Euclidean Distance (SED)	Group/Treatment [Optional]	genotype
	Input Run Order Name [Optional]	run_order
	Additional groups to separate by [Optional]	
	Threshold	0.95
Coefficient of Variation (CV)	Group/Treatment [Optional]	
	CV Cutoff [Optional]	0.1
Principal Components Analysis (PCA)	Group/Treatment [Optional]	
Bland-Altman (BA)	Outlier Cutoff	3
	Sample Flag Cutoff	0.2
	Feature Flag Cutoff	0.05
	Group/Treatment [Optional]	genotype
	Group Name [Optional]	
Generate distribution of features across samples	Group/Treatment [Optional]	genotype

Supplementary Table 2. SECIM Tools workflow, input parameters, and set values used for QC steps on analytical data.

Feature ID	Putative Annotation	Strain	Confidence score
ppm_1_4761	Alanine	VC1265	4
ppm_1_4896			
ppm_2_5027	Alpha-ketoglutaricacid	RB2347	3
ppm_2_237	Aminoadipic Acid*	RB2347	2
ppm_3_2462	Arginine	VC1265	4
ppm_3_2547		VC2524	
ppm_1_6361	Arginine*	AUM2073	4
ppm_1_6525			
ppm_3_1839	Beta-Alanine	RB2347	4
ppm_3_2672	Betaine	VC1265	4
ppm_0_86209	CH3-Lipoprotein	AUM2073	1
ppm_2_5584	Citrate (carbon shift outside threshold criteria)	AUM2073	2
ppm_2_5788		KJ550	
ppm_2_5866		AUM2073	
ppm_2_0689	Glutamic Acid	VC2524	4
ppm_2_0778			
ppm_2_1094		KJ550	
ppm_2_1186			
ppm_2_3474	Glutamic Acid*	RB2347	4
ppm_2_3585			
ppm_2_4498	Glutamine-Unknown*	AUM2073	4
ppm_2_4587			
ppm_2_4673			
ppm_2_4774			
ppm_2_1285	Glutamine/Glutamic Acid*	RB2347	3
ppm_3_5782	Glycerol*	AUM2073	3
ppm_3_9436	Glycero-phosphocholine	RB2347	3
ppm_1_3238	Lactic Acid	VC1265	4
ppm_1_3396			
ppm_1_6686	Leucine*	AUM2073	4
ppm_1_755	Lysine	KJ550	4
ppm_3_0189		VC1265	
ppm_3_029			
ppm_3_0393			
ppm_1_9208	Lysine-Acetic Acid-Arginine*	AUM2073	3
ppm_1_6832	Lysine-Arginine*	KJ550	4
ppm_1_7156		VC1265	
ppm_1_7348			
ppm_7_3231	Phenylalanine	VC1265	4
ppm_7_338			
ppm_7_4387		RB2347	
ppm_3_1231	Phenylalanine*	RB2347	4
ppm_7_3876			
ppm_2_007	Proline (low level)	AUM2073	3
ppm_2_0306		VC2524	
ppm_3_4544	Trehalose	RB2347	4
ppm_3_4704		VC1265	
ppm_5_188		RB2347	
ppm_5_2069		KJ550	
ppm_3_6408	Trehalose-Glycerol*	VC1265	4
ppm_3_6575		RB2347	
ppm_3_8518	Trehalose*	KJ550	4
ppm_3_8699			
ppm_1_591	Unknown 1	AUM2073	n/a
ppm_1_5981			
ppm_1_6058	Unknown 2	KJ550	n/a
ppm_3_2244	Unknown 3	VC1265	n/a
ppm_8_1863	Unknown 4	AUM2073	n/a
ppm_0_94007	Unknown 5*	AUM2073	n/a
ppm_1_9545	Unknown 6*	KJ550	n/a
ppm_2_2768	Unknown 7*	RB2347	n/a
ppm_2_2952	Unknown 8*	RB2347	n/a
ppm_3_7919	Unknown 9*	RB2347	n/a

Supplementary Table 3. List of significant features and respective annotation. FeatureID indicates the chemical shift of each feature. Putative annotation indicates compound name as obtained from COLMAR (86). Strain indicates the corresponding mutant for each feature deemed significantly different (p-value < 0.05). Confidence score defined as 1 to 5, with 5 being the highest. The scale is defined as follows: (1) putatively characterized compound classes or annotated compounds, (2) matched to literature and/or 1D spectra of a reference standard, (3) matched to HSQC, (4) matched to HSQC and validated by HSQC–TOCSY and TOCSY

(COLMARm), and (5) validated by spiking the authentic compound into the sample (82). Unknown has no matches in the COLMAR database. Low level indicates features were low intensity in 2D spectra. Abbreviations: asterisk – feature indicated was found to be overlapped within the 2D spectra, n/a – not applicable.

**Parameters**	**Positive Mode**	**Negative Mode**
	**SIRIUS**	
Instrument	Orbitrap	Orbitrap
Filter by isotope pattern	TRUE	TRUE
MS/MS isotope scorer	IGNORE	IGNORE
MS2 MassDev (ppm)	5	5
Candidates	10	10
Candidates per ion	1	1
Consider only formulas in DBs	TRUE	TRUE
Databases selected:	All but combinatorial	All but combinatorial
Possible ionizations	[M + H]+, [M + K]+, [M + Na]+	[M + Br]-, [M + Cl]-, [M + H]-
Tree timeout	0	0
Compound timeout	0	0
Use heuristic above m/z	300	300
Use heuristic only above m/z	650	650
Elements allows in Molecular Formula	**see below**	**see below**
H	inf	inf
P	inf	inf
C	inf	inf
N	inf	inf
O	inf	inf
	**ZODIAC**	
Considered candidates 300m/z	10	10
Considered candidated 800m/z	50	50
Use 2-step approach	TRUE	TRUE
Edge Threshold	0.95	0.95
Min Local Connections	10	10
Iterations	20,000	20,000
Burn-In	2,000	2,000
Separate Runs	10	10

Supplementary Table 4. Input parameters used for elemental formula generation in SIRIUS (83) and ZODIAC (84).

	RP (+)	HILIC (+)	RP (-)
fold blank	1	1	1
frac qc	1	1	1
alpha*	0.1	0.1	0.1
dmz*	0.005	0.005	0.005
drt*	0.03	0.03	0.03
extracted quantity	height	height	height
num references	150	150	150
ppm	5	5	5
adducts positive	[M+H]+, [M+2H]2+, [M+Na]+, [M+K]+, [M+NH4]+, [M+2Na-H]+, [2M+H]+, [2M+2H]2+, [2M+H+Na]2+, [2M+Na]+, [2M+2Na-H]+, [M+2H-NH3]2+, [M+H-H2O]+, [M+H-H2O]+, [M+2H-H2O]2+, [M+3H]3+, [M+CH3COONa+H]+, [M+CH3COONa+Na]+, [M+CH3COONa+NH4]+	[M+H]+, [M+2H]2+, [M+Na]+, [M+K]+, [M+NH4]+, [M+2Na-H]+, [2M+H]+, [2M+2H]2+, [2M+H+Na]2+, [2M+Na]+, [2M+2Na-H]+, [M+2H-NH3]2+, [M+H-H2O]+, [M+H-H2O]+, [M+2H-H2O]2+, [M+3H]3+, [M+CH3COONa+H]+, [M+CH3COONa+Na]+, [M+CH3COONa+NH4]+	[M-H]-, [M-2H]2-, [M-2H+Na]-, [M-H+Cl]2-, [M-2H+K]-, [M+Cl]-, [2M-H]-, [2M-2H+Na]-, [2M-H+Cl]2-, [2M-2H+K]-, [2M+Cl]-, [M-H-H2O]-
dmz	0.005	0.005	0.005
main adducts positive	[M+H]+, [M+2H]2+, [M+Na]+, [M+NH4]+	[M+H]+, [M+2H]2+, [M+Na]+, [M+NH4]+	[M-H]-, [M+Cl]-, [2M-H]-
max charge	3	3	3
max isotopes	4	4	4
min filter	2	2	2
num files	100	100	100
polarity	positive	positive	negative
ppm	5	5	5
files used	12	12	12
need optimization	TRUE	TRUE	TRUE
noise threshold	1000	1000	1000
num iterations	5	5	5
number of points	30	30	50
ms1	gap-filled data matrix	gap-filled data matrix	gap-filled data matrix
ms2	fused mgf	fused mgf	fused mgf
algorithm	ADAP	ADAP	ADAP
noise level ms1	1000	1000	1000
noise level ms2	1000	1000	1000
SN*	4.57	17.38	15.6
coefficient area threshold*	57.94	108.56	39.05
ms2 mz tol	0.005	0.005	0.005
ms2 rt tol	0.1	0.1	0.1
noise level	10000	10000	10000
peak width (min/max)*	0.018 / 0.718	0.030 / 0.434	0.083 / 0.581
rt wavelet (min/max)*	0.001 / 0.081	0.002/ 0.110	0.012 / 0.103
peaktable filter	absolute intensity top 30000	absolute intensity top 30000	absolute intensity top 30000
dmz*	0.005	0.004	0.002
min scan*	6	4	4
ppm*	6	12	6

*value determine via SLAW optimization

Supplementary Table 5. Pre-processing steps, input parameters, and set values used for LC-MS data are listed in the execution order**.**

Study Group	Significant features unique to meta-study model				
	NMR		LC-MS		
	Non-polar	Polar	RP +	RP -	HILIC +
NS*	23	29	133	8	6
UGT	11	12	61	6	5
CMM	11	20	179	13	8

** excluding natural strain N2 (see supplemental information)*

Supplementary Table 6. Features that were not significant in any of the individual meta-strain model comparisons but are significant when compared via the meta-study model.
